# High-Resolution Copy-Number Variation Map Reflects Human Olfactory Receptor Diversity and Evolution

**DOI:** 10.1371/journal.pgen.1000249

**Published:** 2008-11-07

**Authors:** Yehudit Hasin, Tsviya Olender, Miriam Khen, Claudia Gonzaga-Jauregui, Philip M. Kim, Alexander Eckehart Urban, Michael Snyder, Mark B. Gerstein, Doron Lancet, Jan O. Korbel

**Affiliations:** 1 Department of Molecular Genetics, Weizmann Institute of Science, Rehovot, Israel; 2 Centro de Ciencias Genómicas, Universidad Nacional Autónoma de México, Cuernavaca, Morelos, México; 3 Department of Molecular Biophysics and Biochemistry, Yale University, New Haven, Connecticut, United States of America; 4 Department of Molecular, Cellular, and Developmental Biology, Yale University, New Haven, Connecticut, United States of America; 5 Program in Computational Biology and Bioinformatics, Yale University, New Haven, Connecticut, United States of America; 6 Department of Computer Science, Yale University, New Haven, Connecticut, United States of America; 7 Structural and Computational Biology Unit, European Molecular Biology Laboratory, Heidelberg, Germany; University of Minnesota, United States of America

## Abstract

Olfactory receptors (ORs), which are involved in odorant recognition, form the largest mammalian protein superfamily. The genomic content of OR genes is considerably reduced in humans, as reflected by the relatively small repertoire size and the high fraction (∼55%) of human pseudogenes. Since several recent low-resolution surveys suggested that OR genomic loci are frequently affected by copy-number variants (CNVs), we hypothesized that CNVs may play an important role in the evolution of the human olfactory repertoire. We used high-resolution oligonucleotide tiling microarrays to detect CNVs across 851 OR gene and pseudogene loci. Examining genomic DNA from 25 individuals with ancestry from three populations, we identified 93 OR gene loci and 151 pseudogene loci affected by CNVs, generating a mosaic of OR dosages across persons. Our data suggest that ∼50% of the CNVs involve more than one OR, with the largest CNV spanning 11 loci. In contrast to earlier reports, we observe that CNVs are more frequent among OR pseudogenes than among intact genes, presumably due to both selective constraints and CNV formation biases. Furthermore, our results show an enrichment of CNVs among ORs with a close human paralog or lacking a one-to-one ortholog in chimpanzee. Interestingly, among the latter we observed an enrichment in CNV losses over gains, a finding potentially related to the known diminution of the human OR repertoire. Quantitative PCR experiments performed for 122 sampled ORs agreed well with the microarray results and uncovered 23 additional CNVs. Importantly, these experiments allowed us to uncover nine common deletion alleles that affect 15 OR genes and five pseudogenes. Comparison to the chimpanzee reference genome revealed that all of the deletion alleles are human derived, therefore indicating a profound effect of human-specific deletions on the individual OR gene content. Furthermore, these deletion alleles may be used in future genetic association studies of olfactory inter-individual differences.

## Author Summary

Copy-number variants (CNVs) are deletions and duplications of DNA segments, responsible for most of the genome variation in mammals. To help elucidate the impact of CNVs on evolution and function, we provide a high-resolution CNV map of the largest gene superfamily in humans, i.e., the olfactory receptor (OR) gene superfamily. Our map reveals twice as many olfactory CNVs per person than previously reported, indicating considerable OR dosage variations in humans. In particular, our findings indicate that CNVs are specifically enriched among evolutionary “young” ORs, some of which originated following the human-chimpanzee split, implying that CNVs may play an important role in the gene-birth and gene-loss processes that continuously shape the human OR repertoire. Furthermore, we describe 15 OR gene loci showing frequent human-specific deletion alleles. Additionally, we present evidence for a recent non-allelic homologous recombination event involving a pair of OR genes, forming a novel fusion OR that may harbor novel odorant-binding properties. Such events may potentially relate to individual functional “holes” in the human smell-detection repertoire, and future studies will address the specific chemosensory impact of our genomic variation map.

## Introduction

Olfaction, the sense of smell, is characterized by the remarkable ability to detect and discriminate millions of odorous compounds. It contributes significantly to our perception of the environment and to our quality of life [1]. At the molecular level, olfaction is mediated by a conserved signal transduction cascade, which is initiated by the binding of odorants to specific G-protein coupled receptors, known as olfactory receptors (ORs) [2],[3]. The human OR repertoire is comprised of 851 genes and pseudogenes, organized in clusters on almost every chromosome [4]. During human evolution these gene clusters underwent dynamic processes of expansion, diversification and duplication as well as diminution and pseudogenization [5], processes which may be still ongoing. Since members of the human species depend on their sense of smell to a lesser degree than other mammals, their OR repertoire has undergone an accelerated process of pseudogenization, resulting in functional inactivation of more than 50% of the ORs by frame-disrupting mutations. Furthermore, the lower degree of purifying selection in human olfaction also appears to result in enhanced inter-individual genome diversity, e.g. the prevalence of high-frequency inactivating single nucleotide polymorphisms (SNPs), i.e. segregating pseudogenes [6]. Thus, the human OR repertoire serves as an interesting model for genome evolution and variability.

Human olfactory perception exhibits considerable phenotypic variation, for which a genetic basis has been proposed [7]–[10]. Recently two studies associated common SNPs affecting OR genes *OR7D4* and *OR11H7P* to human sensitivity and perception of the respective odorants androstenone and isovaleric acid [11],[12]. These findings support a relationship between genotypic and phenotypic variability in human olfaction.

However, recent results indicate that most of the variation in the human genome is not accounted for by SNPs. Genome structural variants, usually defined as kilobase (kb) to megabase (Mb) deletions, duplications, insertions, and inversions, emerge as responsible for the majority of variable base-pairs among individuals [13]–[15] and may be a main basis for phenotypic differences [16]. Structural variants affecting the copy-number of a ≥1 kb genomic region (e.g. through deletion or duplication) are widely referred to as Copy Number Variants (CNVs; [17]). Several genome wide surveys have recently reported a prevalence of OR genes among CNVs [13],[14],[18],[19], suggesting an impact of CNVs on the individual OR gene content. Moreover, recent reviews have suggested that CNVs have an impact on the genome in longer evolutionary terms by facilitating the expansion or diminution of gene families [20],[21]. However, these genome-wide surveys did not focus on ORs specifically, and were either carried out at low-resolution, suitable for genomic clusters of ORs rather than for single OR loci, or considered few (i.e., 8) individuals. A gene-level resolution survey of copy-number variability in the largest mammalian gene superfamily should thus serve as an excellent model for studying long-term effects of CNVs on the genome. Furthermore, such a survey may help identifying candidates for examining potential phenotypic consequences of CNVs on smell perception.

Here, we report a high-resolution analysis of CNVs affecting the human OR repertoire. We use custom high-resolution oligonucleotide tiling microarrays to study OR gene copy-number variation in 25 individuals with ancestry in three human populations, and report that about a fourth of all OR genes are commonly affected by CNVs. In addition, we find that CNVs affecting ORs are more complex than previously appreciated. Furthermore, evolutionarily “young” ORs, as well as OR pseudogenes, show a strong tendency for copy-number variation. In addition, using confirmatory quantitative PCR (qPCR), we report a total of 15 OR gene loci with an appreciable prevalence of homozygous deletion genotypes in humans. These deletions may provide an additional, CNV-based, genotypic basis for variations in human olfactory perception.

## Results

### A High-Resolution Map of CNVs Affecting OR Genes and Pseudogenes

**Figure 1:**
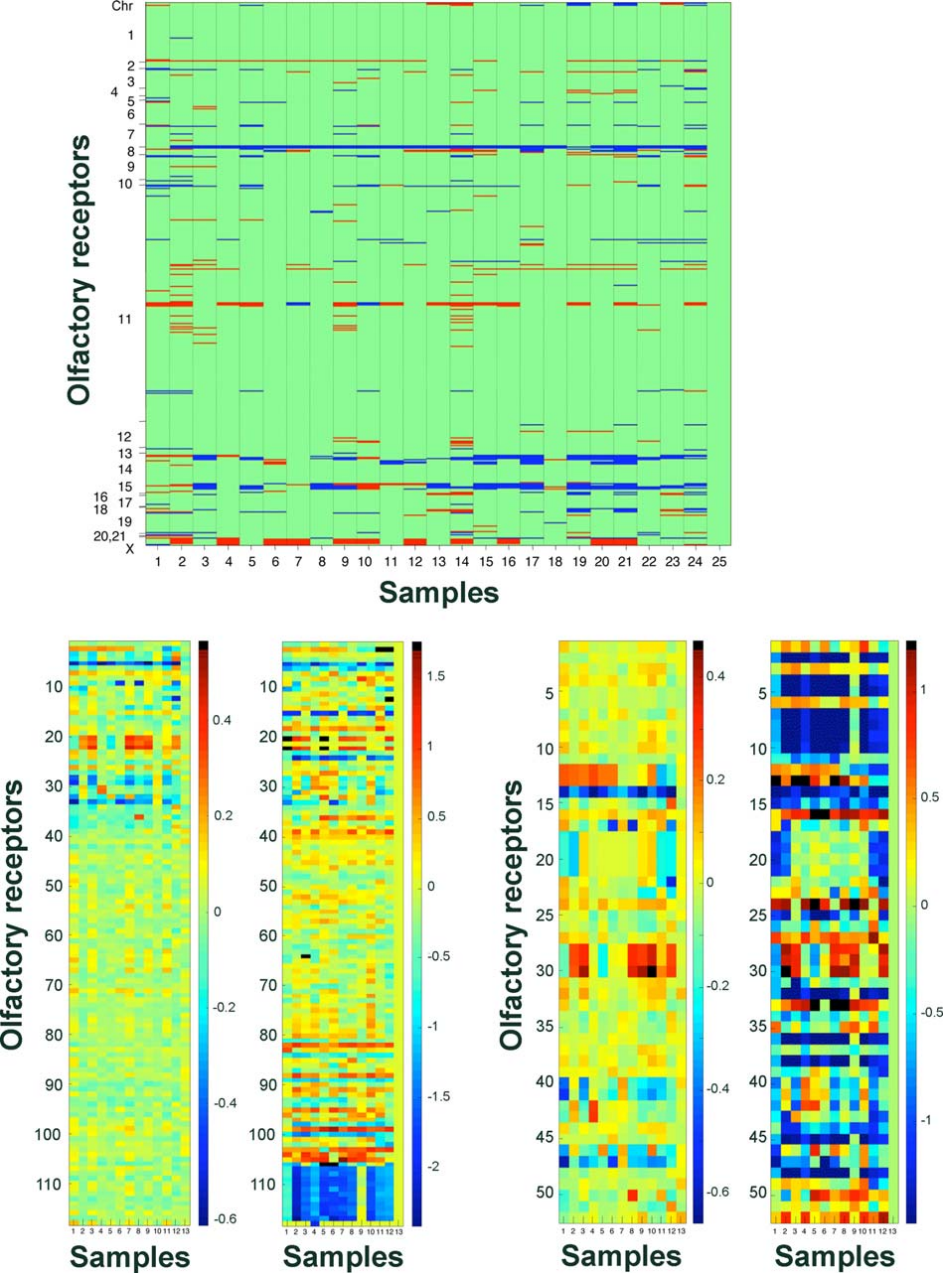
A high-resolution map of CNVs in the human OR repertoire. A) CNV map for OR loci based on high-resolution oligonucleotide tiling arrays. 851 ORs are ordered according to their location along the chromosomes, as indicated on the left; rows represent genes, columns are individuals; gains are shown in red, losses in blue and un-changed dosage in green (calls were made relative to the male reference individual NA19154). Note that the non-uniform genomic distribution of ORs results in an unbalanced representation of chromosomes in panel A. Also, note that ‘gains’ on chromosome X do not represent CNVs but refer to the expected male/female dosage difference. CNV calls are given for all 25 individuals (i.e. a self-*vs.*-self-replicate of NA19154 was included as a control; see Methods). Due to the resolution of the figure single CNVs may not be visible (all events are given in Table S2). Samples appear in the following order (1–25); NA10851, NA11997, NA12003, NA12004, NA12005, NA12006, NA12246, NA12248, NA12865, NA15510, NA18501, NA18502, NA18504, NA18505, NA18506, NA18508, NA18611, NA18856, NA18945, NA18946, NA18972, NA19103, NA19128, NA19141, NA19154. B) qPCR and microarray measurements of 122 OR loci for 13 individuals. The right panel represents qPCR results, and the left panel the corresponding microarray measurements (i.e. the measure *R*; see Methods). Sixty of the 122 ORs were tested in 13 individuals, thus only data for these samples is shown (for the full dataset, see Table S1). OR loci were sorted based on copy-number variability as assessed with our microarrays; the top 40 rows represent genes categorized as CNVs by microarrays; the lower part refers to loci not scored as a CNVs with the arrays, but scored as CNVs by qPCR (see Table 2). qPCR data was normalized relative to NA19154, and inverted (values multiplied with −1) to fit to the microarray scale. *OR2BH1P* and *OR9G1* showed homozygous deletion in the reference individual, thus the qPCR values of these ORs were not normalized. Relative intensities are color coded, as indicated by the color scales. Homozygously deleted OR alleles are shown in the right panel in black. Samples appear in the following order; NA12003, NA12004, NA12005, NA12006, NA12246, NA12248, NA12865, NA18504, NA18508, NA18856, NA19103, NA19141, NA19154. C) qPCR-measurements and array (*R*) measures for the 56 most variable OR loci. The most variable OR loci were selected based on variance in qPCR results. Representation and sample order is as in panel B.

Analyzing the microarray results we identified OR loci with median normalized microarray log_2_-intensity ratios *R* that significantly deviated from expected measures, and were thus scored as CNVs (see Methods). Altogether, we observed 1301 CNV events (gains and losses) affecting loci harboring intact OR genes and pseudogenes in the 25 individuals analyzed (Figure 1A). Notably, due to the comparative nature of our analysis, this results in 24, rather than 25, sets of CNVs. Of the 851 OR loci interrogated, 244 ORs (28%, including 93 intact genes and 151 pseudogenes) were observed to be affected by CNVs (Tables 1, S1). Next, we examined to what extent CNVs affect the individual genomic OR content. On average, we observed CNV events in 22 OR genes and 32 OR pseudogenes per sample (mean number of CNV events per sample; see Methods). The vast majority of these probably represent true positives, as we generally estimate a false-positive rate of less than 4% for CNV assignments based on a control (self-*vs.*-self hybridization; see Methods). Interestingly, meta-analysis of lower resolution data performed by Nozawa *et al.* [19] indicated a mean difference of only 10.9 intact OR genes and 11.3 OR pseudogenes between individuals. Our considerably higher numbers are presumably explained by the fact that many CNVs detected in our study are below the resolution of the CNV-detection methods that were available to Nozawa *et al.* (see e.g. discussions in recent studies using high-resolution mapping approaches [14],[15],[29]).

**Table 1:** Summary of array results for OR gene and pseudogene loci and comparison with DGV.

	*Intact*	*Pseudogenes*	Total
**# Genes in array**	385	466	**851**
**# Genes that show gains**	68	122	**190**
**# Genes that show losses**	53	82	**135**
**# Variable genes**	93 (24%)	151 (32%)	**244 (28%)**
**# Genes in**			
**New CNVs**	42	68	**110**
**Confirmed CNVs**	51	83	**134**
**Undetected CNVs**	106	118	**224**
**Confirmed not-variable**	188	195	**383**

Copy-number variable loci are OR loci with *R*–measures (median normalized microarray intensity log_2_-ratio across an OR locus) falling beyond the cutoff *C* = |0.18| (see Methods). ORs were considered as copy-number variable if a gain or loss was identified in at least one individual. The variability status of each locus was compared to the Database of Genomic Variants (DGV). “New CNV”: CNV identified by us, not in DGV; “Confirmed CNV”: CNV identified by us, present in DGV; “Undetected CNV”: present in DGV, not identified in our panel of individuals; “Confirmed not variable”: was not identified as being copy-number variable by us, and is not reported in DGV.

A more detailed view of the microarray results was generated for two subsets of the OR repertoire. One (Figure 1B, left panel) encompasses the OR gene subset for which qPCR has been performed. The second, an even stronger, zoom view is afforded by the left panel of Figure 1C, where the most variable 25 loci, with 56 OR genes, are shown. Both views highlight the fact that CNVs create unique mosaics of OR dosages across individuals that might contribute to the functional individuality of the human nose. Furthermore, the qPCR results led us to detect 23 additional CNVs that were not detected by the microarrays and were added to our map. Fifteen of these intersect with CNVs reported in the Database of Genomic Variants (DGV; http://projects.tcag.ca/variation/; see Table 2), which corroborates our qPCR results. Such a false-negative rate was not unexpected for the array-based CNV-calls, since microarrays have been reported to miss some true-positive CNVs [13]–[14].

**Table 2:** CNVs detected in qPCR experiments that displayed little variability in microarray experiments.

OR locus	qPCR-variance	variance of *R*	Status in DGV
OR10AG1	0.693519007	0.002244359	CNV
OR10J3	0.227390436	0.000961263	NR
OR10J5	0.22072615	0.00106244	NR
OR10Z1	0.573520272	0.001973186	NR
OR1A2	0.176006731	0.000929803	CNV
OR1G1	0.418577965	0.002270532	CNV
OR2AJ1	0.414815785	0.002147011	CNV
OR2B11	0.396684535	0.005136635	CNV
OR2G3	0.396707452	0.001385149	CNV
OR2L13	0.511584535	0.002920064	CNV
OR2T29	0.150669223	0.003118704	CNV
OR2T6	0.61481576	0.000332848	CNV
OR4A45P	0.226808226	0.003287325	CNV
OR4C13	0.494025825	0.002679713	CNV
OR4E2	0.387122516	0.001315008	CNV
OR4K14	0.162403526	0.001719849	CNV
OR5AK2	0.261028206	0.003661852	NR
OR6C2	0.410398478	0.003290131	CNV
OR6C4	0.193139103	0.001050314	CNV
OR6K2	0.173307692	0.002568683	NR
OR6Q1	0.240047903	0.002727374	NR
OR6Y1	0.162319311	0.002412609	NR
OR9G1	0.295386696	0.001038619	NR

Twenty-three ORs were found to be variable in qPCR experiments (beyond a conservative cutoff for the variance of >0.15), but not in the microarrays. NR: not previously reported.

The high resolution of our approach also allowed us to more precisely predict the location of CNV breakpoints from microarrays. To this end we used the microarray-data based *BreakPtr* algorithm [23] to identify instances where the breakpoints of CNVs are located within an OR locus (see Methods). Disrupted/split loci were detected for 88 ORs, suggesting a surprising complexity of CNV genotypes (Table S3). For eight of these loci our data suggest recurrent CNV formation events, since for different samples distinct breakpoint locations were predicted (Table S3). While most CNV breakpoints do not disrupt the ∼900 bp OR coding regions, the *BreakPtr* algorithm predicts disruption of coding regions of four specific OR loci (*OR4C16*, *OR1M1*, *OR6B2*, and *OR4N2*; see Table S3). Although further experiments will be necessary to validate these predicted coding region disruptions, additional sequence analyses described below indicate that OR coding sequence may be directly involved in CNV formation, e.g. leading to novel OR fusion genes.

### Validation of Identified CNVs

We used three different methods for CNV validation, i.e. (i) comparing our CNV-calls to previously reported CNVs from DGV [30], (ii) performing quantitative PCR (qPCR), and (iii) performing conventional PCR. Initially, when comparing our results to DGV, we found that in total, 134 (55%) copy-number variable OR loci intersected with previously described CNVs (Table 1). Our capacity to discover a large number of new variable loci, despite the fact that relatively few individuals were examined when compared to the multiple studies contributing to DGV, stresses the sensitivity and overall value of our high-resolution CNV map.

**Figure 2:**
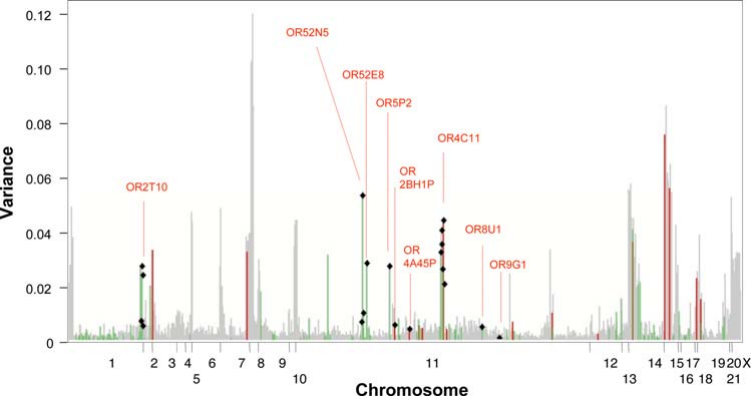
Copy-number variability expressed as variance of experimental measures. Variance in array measurements is indicated along OR loci, with loci arranged according to genomic coordinates. The variance of individual array measurements for each OR is plotted in grey. Array variance of ORs that were assayed by qPCR is color-coded; green: OR genes; red: OR pseudogenes. Black squares indicate ORs listed in Table 3; representative ORs from each cluster are indicated by red doted lines.

Subsequently, we validated CNVs by performing qPCR on 122 OR loci (104 ORgenes and 18 pseudogenes), which were selected to represent both high and low variability loci (Figure 2, S2) and which included similar amounts of novel and already known CNVs. Sixty validation experiments were carried out with a panel of only 13 samples, while the other 62 experiments were performed in 23 samples. We initially compared individual microarray intensities (normalized log_2_-intensity ratios) to qPCR outcomes (normalized Cp-values). In general, qPCR results revealed an acceptable correlation to the normalized microarray values (Figure S3A, S3B), with the exception of a small group of 23 OR loci that displayed considerable qPCR inter-subject variability despite low-variability microarray values (Figure S3A, S3B ). We added these 23 cases to our list of copy-number variable OR loci (Table 2; Figure S4).

Next, we examined the rate at which CNV calls were validated by qPCR (qPCR results were therefore normalized relative to reference individual NA19154): 87% of all CNVs tested yielded negatively correlating Cp-values, as expected, indicating successful validation, with similar success rates for gains and losses. In the majority of cases, rounded absolute qPCR-measures were equal to Cp = 1, in line with an abundance of simple gains and losses (i.e. heterozygous deletions and duplications). Taken together, most CNV calls can be validated by qPCR, demonstrating a very reasonable specificity of our platform.

Finally, for two cases (*deletions I* and *II*, Table 3), we validated qPCR results that indicated homozygous deletions, i.e. such that consistently failed in specific DNA samples, by conventional PCR with an additional set of primers (Figure S5). For both deletion alleles, two positive and two negative samples were tested. Standard PCR confirmed our results in both cases, validating the presence of homozygous *deletion I* in NA12003 and NA12246, and homozygous *deletion II* in NA19103 and NA19141.

**Table 3:** Summary of deletion alleles followed up in detail.

#.	# ORs deleted	ORs deleted	Location	Start	End	Length (kb)	Former status	Ref.	# of homozygously deleted samples in qPCR	Estimated deletion allele** frequency	Human derived allele
I	6	OR4C11 OR4P4 OR4S2 OR4C6 OR4V1P OR4P1P	11q11	55,124,730	55,207,364	82.6	Deletion	Watson genome	3	0.36	deletion
II	4	OR2T34 OR2T10 OR2T11 OR2T35	1q44	246,794,522	246,875,051	80.5	Deletion	Watson genome	2	0.29	deletion
III	2	OR8U8 OR8U9	11q11	53,483,709	53,491,314	7.6	NR	-	0*	0.09	deletion
IV	1	OR2BH1P	11p14.1	28,962,961	28,970,373	7.4	CNV-loss	DGV	8	0.59	deletion
V	1	OR4A45P	11p11.2	48,557,433	48,560,858	3.4	CNV-loss	DGV	5	0.46	deletion
VI	3	OR56B2P OR52N5 OR52N1	11p15.4	5,740,460	5,766,804	26.3	CNV-loss	DGV	1	0.2	deletion
VII	1	OR52E8	11p15.4	5,828,206	5,839,952	11.7	CNV-loss	DGV	1	0.2	deletion
VIII	1	OR9G1	11q11	53819045*	53830192*	ND	NR	-	3	0.36	deletion
IX	1	OR5P2	11p15.4	7,767,796	7,792,963	25.2	CNV	DGV	1	0.2	deletion

The table summarizes losses affecting OR loci for which we sought for homozygously deleted individuals using qPCR. Abbreviations: NR: not reported previously; DGV: intersecting variant previously reported in the Database of Genomic Variants (http://projects.tcag.ca/variation). “Former status” indicates as to whether this locus was reported as a “deletion”, or “CNV”, in previously published data sets. Boundaries of an event were here taken from the respective (confirmatory) data source (except for the novel *deletions III* and *VIII*). *For *deletion III*, 4 heterozygotes (and no homozygous deletion) were identified by combinations of allele-specific qPCR reactions.**For all deletions, except *deletion III*, allele frequencies were estimated as square root of proportion of homozygously deleted individuals. Frequency of *deletion III* allele was estimated as the number of deletion alleles (n = 4) out of total number of chromosomes tested (n = 46).

### Genomic Distribution of CNVs

CNVs affecting OR loci are strongly non-uniformly distributed, causing a clustering of CNVs into ‘hotspots’, and an associated clustering of regions with high variance in normalized microarray intensities (see Figures 1A, 2). Accordingly, there is a strong correlation (0.8, *P*-value = 10^−18^) between the variance (based on measure *R*) of a variable OR to the variance of adjacently located ORs (Figure S6A). Some of the clustering can be explained with an enrichment of CNVs near telomeres and centromeres (Figure S6B), consistent with observations from a previous report [13]. Genomic CNV formation biases are a plausible explanation for the clustering. Furthermore, CNV size (e.g. CNVs affecting multiple ORs) and biases in evolutionary selection are likely also responsible for the observed non-uniform distribution in our data (see below).

We analyzed apparent CNV ‘hotspots’ in more detail, and observed that the 244 variable OR loci were clustered into 149 extended genomic *blocks* displaying a common pattern of variability (Figure S7). To examine whether observed blocks of high copy-number variability actually behave as single (large) CNVs we performed an analysis addressing the correlation of cross-individual patterns within each of these blocks (Figure S6C). Indeed, the analysis identified 31 copy-number blocks containing 105 OR loci with a Pearson correlation coefficient >0.8 in cross-individual normalized microarray measures *R*, indicating that the blocks likely correspond to a single, large CNV. The largest such block is located on chromosome 14 and contains 11 OR loci. On the other hand, this suggests that many of the remaining candidate hotspots may underlie several independent (i.e. smaller) CNVs occurring in close proximity. However, although large CNVs (such that span more than one OR locus) are relatively rare (involving 43 out of OR 149 loci only), they contribute to our inter-individual variability to approximately the same extent as small CNVs. Moreover, the multi-locus CNVs are important from a functional aspect, as large CNVs that affect several ORs simultaneously are more likely to result in detectable phenotypes than small ones.

### Evolutionary Selection and Formation Bias as Contributors to Copy-Number Variation in the OR Repertoire

**Figure 3:**
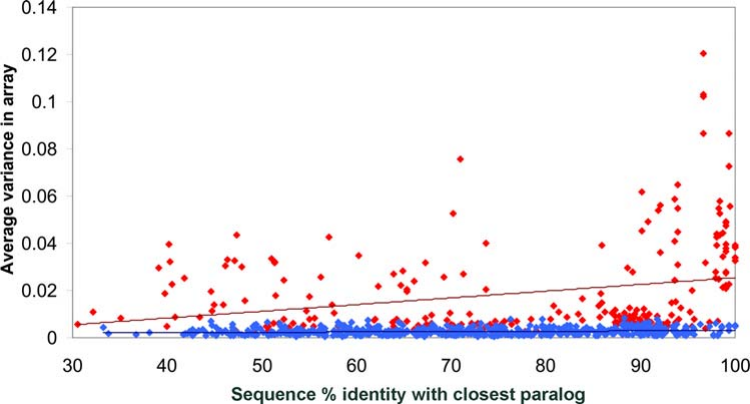
Correlation of OR copy-number variability with paralog-similarity. Red and blue dots indicate copy-number variable and non-variable ORs, respectively (copy-number variability is expressed in terms of the measure *R*, see Methods, which we found to correlate well with gene dosage). Percentage DNA sequence identity (“% identity”) to the closest paralog in the human genome is plotted versus the array-based (i.e., *R*-measure-based) variance. Correlation for ORs affected by CNVs is C = 0.26 (P_value_ = 10^−5^), whereas for non-variable ORs it is C = 0.15 (P_value_ = 10^−4^). Linear regression fits for each dataset are indicated with red and blue dashed lines, respectively.

**Figure 4:**
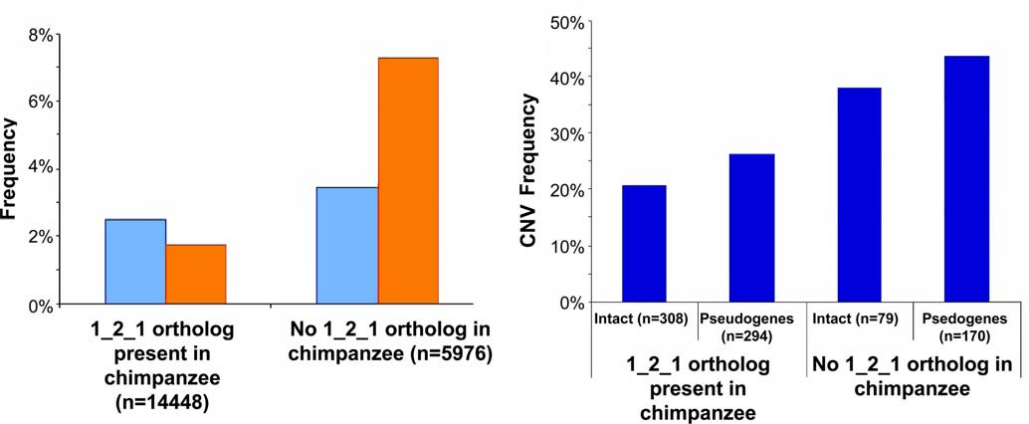
CNVs preferentially affect ORs lacking unambiguous one-to-one orthologs in the chimpanzee genome. A) Gains and losses of OR loci were called using our microarrays (see Methods). Gains are shown in blue; losses in orange; *n* is the number of total calls considered (24 samples multiplied by the number of genes in each category). OR loci with a one-to-one (“1-2-1”) ortholog in the chimpanzee genome are significantly (P_value_<0.001; Mann-Whitney U test) less often affected by CNVs than loci lacking a 1-2-1 ortholog. B) Frequencies of CNV loci are given separately for intact OR genes and pseudogenes in each of the evolutionary classes. “Frequency”: relative frequency of being called a CNV for a set.

Our high-resolution map of CNVs affecting OR genes further enabled us to address questions relating to the evolution of the OR repertoire. We first tested whether the evolutionary age of an OR gene is correlated with its propensity to be affected by a CNV. Interestingly, we found that ORs with a closely related paralog in the human genome, evaluated using the level of sequence identity as a measure, are significantly more likely to be affected by CNVs than ORs lacking a closely related paralog (Figure 3). In other words, evolutionarily “younger” ORs tend to be more frequently affected by CNVs than more “ancient” ORs. To confirm this trend using a different approach to classify OR genes into “young” and “ancient” we used one-to-one orthology relationships with the chimpanzee genome and categorized OR genes into “young” if they lacked a one-to-one ortholog and “ancient” in the case of unambiguous orthology: indeed, we found that OR genes that recently exhibited a duplication/loss event in the human, or the chimpanzee genome (i.e. such genes lacking one-to-one orthologs between human and chimp), are significantly more likely to be affected by CNVs than OR genes with unambiguous one-to-one orthologs (P_value_<0.001, Figure 4A). We note that these findings are compatible with a suggested general model of copy-number variation as an evolutionary basis of paralog birth [20],[21], whereby novel paralogs, manifested as CNVs, may later become fixed in the population.

We further note that the enrichment of CNVs in “younger” OR genes may be due to two reasons: First, the higher number of CNVs observed in ”younger” ORs may be due to decreased selective pressures in “young” ORs compared to “ancient” ORs (see below). Second, different CNV formation biases acting on genomic regions harboring “young” and “ancient” ORs may be responsible. We first evaluated the impact of CNV formation bias through testing whether pairs of tandemly oriented segmental duplications (SDs; [31]) – known mediators of CNV *de novo* formation through induction of non-allelic homologous recombination (NAHR) – are enriched among the ORs affected by CNVs. When performed at single gene resolution, this analysis yielded inconclusive results, which may be due to a confounding positional bias caused by the fact that neighboring OR loci are often located in close vicinity in the genome, forming genomic clusters (see Text S1). We thus examined the potential impact of NAHR also at the level of 135 genomic clusters of OR loci, which are listed in the HORDE database (http://bioportal.weizmann.ac.il/HORDE/index.html), and observed a significant and robust enrichment (with P-values<0.01) of SD-pairs among highly copy-number variable clusters compared to clusters for which no single CNV has been observed in our study (see Text S1 for details and Figure S8). Thus, our data indicate that NAHR has likely biased the distribution of CNVs within the OR repertoire.

Next, we tested whether, and to what extent, evolutionary selection may influence CNVs affecting the OR repertoire. In this regard, we first revisited the recent report that OR pseudogenes are equally likely to be affected by CNVs as OR genes, a finding suggesting a neutral evolution of OR copy number variation [19]. In particular, we compared CNVs in OR genes and pseudogenes, and found that OR pseudogenes are significantly more likely to be affected by CNVs than OR genes (Table 1, P_value_ = 0.007, χ^2^ statistic = 7.38, DF = 1). A consistent and significant signal was also observed when carrying out the analysis at the level of microarray probes, either only for the coding regions or for the adjacent non-coding regions (see supplementary Text S1). This may indicate that stronger evolutionary constraints act on OR genes than on pseudogenes. Additionally, we analyzed the frequency of CNVs among OR genes and pseudogenes separately for the “young” and “ancient” ORs (Figure 4B). This analysis showed that the difference between “young” and “ancient” ORs is greater than the difference between OR genes and pseudogenes. Nonetheless, in both groups OR genes were less prone to CNV formation than pseudogenes, indicating a mixed contribution of formation bias and selection (see below).

**Figure 5:**
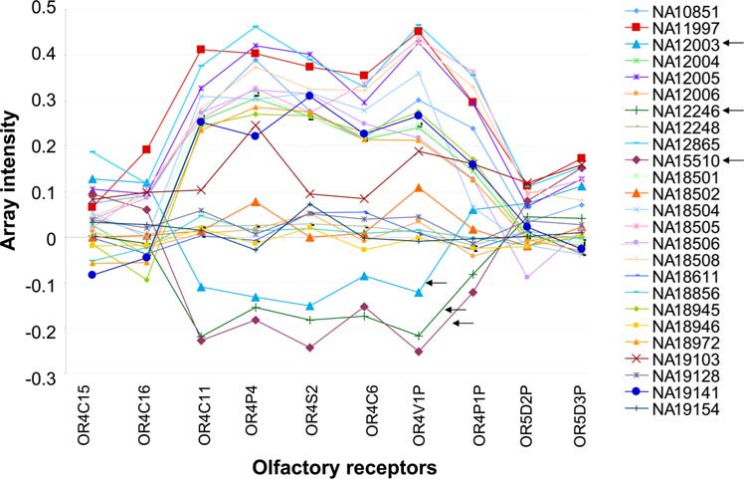
Zoom into a bi-allelic CNV affecting OR4C11. Plot depicting median normalized log_2_-ratios of microarray intensities for OR loci affected by *deletion I* (chr11: 55127497–55238834), a bi-allelic CNV. Each individual is color-coded as indicated in the legend shown to the right. Black arrows indicate samples that consistently failed to produce results in the qPCR and standard PCR assays, indicating a potential homozygous deletion.

Having established that “young” ORs are more prone to CNVs than the “ancient” ones, we further addressed which types of CNVs, i.e. gains or losses, are most common in the two OR groups. Interestingly, for both “ancient” and “young” ORs we observed significant imbalances between gains and losses (Figure 5A, P_value_ = 0.006, χ^2^ statistic = 10.3, DF = 2 and P_value_ = 0, χ^2^ statistic = 43.8, DF = 2, respectively). Notably, the two groups exhibited an opposite over-all trend, i.e. “ancient” ORs displayed significantly more gains than losses and “young” ORs showed significantly more losses than gains. As we arbitrarily picked a reference individual in our study, we also tested whether the observed trend was robust if neglecting the reference, by looking for ORs that exhibited only one type of CNV – gain or loss – in at least 50% of the samples (Text S1 material). Although this analysis revealed that some of the reported events are likely attributable to rare alleles in the reference individual, the trend of opposite balances between gains and losses in the two groups remained significant (P_value_ = 0.004, χ^2^ statistic = 11.1, DF = 2).

### Potential Functional Impact of CNVs Affecting OR Genes

CNVs affecting OR loci may encompass deletions, duplications or more complex allelic structures. In this realm, homozygosity for deletion alleles is the CNV-related genotype that is most likely to cause a direct olfactory phenotypic effect. This is because, with some exceptions, OR gene disruption is likely to be recessive, i.e. only absence of the functional gene product will result in an observable phenotype. In particular, a dosage effect stemming from a different number of active OR copies is not expected due to clonal exclusion, whereby only one paralog is expressed in any given sensory neuron [32],[33]. Thus, we particularly focused on the identification of frequent deletion alleles, which may lead to common differences in smell perception between individuals.

While carrying out qPCR-based validation experiments, for several OR loci we immediately identified samples that consistently failed to amplify during qPCR, suggesting a potential homozygous deletion of the OR locus (Table 3). For example, two samples (NA12003 and NA12246) consistently failed to amplify neighboring ORs from a specific locus on chromosome 11 (Figure S5). In particular, this variation was found to harbor a genomic region of 82 kb involving 4 OR genes and 2 OR pseudogenes. The distribution of median normalized microarray intensity log_2_-ratios (measure *R*) of OR loci in this region (Figure 5) is consistent with a bi-allelic CNV and with dosage levels corresponding to 0, 1, or 2 copies. Furthermore, PCR with uniquely designed primers for ORs in that region yielded no product for NA12003 and NA12246 and bands of expected size in positive controls (NA12004 and NA19141), thus supporting both the microarray and the qPCR results (Figure S5).

We note that a subset of common OR deletions may not be tractable with the approach presented here, as they were deleted from the individual(s) contributing to the human reference genome, which we used to design our high-resolution arrays. Thus, to identify additional potential deletions affecting OR genes, we further searched for discrepancies between the human genome reference assembly and other human genome sequencing projects. In particular, we searched for OR genes that reside in regions of disagreement between the reference genome and publicly available data from the initial Celera human genome assembly [34], Craig Venter's genome [28], James Watson's genome [35], and fosmid clones which are not part of the current reference assembly. Altogether, we identified three OR genes, *OR8U8*, *OR8U9* and *OR9G9*, which are not present in the current reference assembly. The first two present an interesting case, whereby two intact OR genes (*OR8U8* and *OR8U9*) appear to have recently fused through NAHR, which led to the formation of a chimeric OR gene (*OR8U1*; Figure S9). Moreover, sequence comparison between *OR8U8*, *OR8U9*, and *OR8U1* identified a plausible NAHR recombination region, i.e. a 119 bp interval (bp 472–590 in Figure S9), which displays 100% DNA sequence identity between the three genes. qPCR with allele specific primers, designed at the interface of the recombination region, identified 4 individuals heterozygous for the deletion (Table 3). A similar case, where the recombination between *OR51A2* and *OR51A4* results in a new gene encoding an amino acid sequence identical to OR51A4 and upstream regions from the *OR51A2* gene was reported previously [14]. Together, these observations indicate that ORs themselves, rather than their genomic environment, may frequently promote CNV formation through NAHR. In the third case, *OR9G9*, although absent from the reference genome, was identified in all individuals tested by qPCR. However, its closest paralog – *OR9G1* was homozygously deleted in 3 out of 23 individuals tested.

Altogether, fosmids and the individual genomes of Craig Venter and James Watson supplied additional support for 3 deletion loci (*deletions I,II* and *VI*; Table 3) identified by qPCR (Table 3). We further followed up 9 regions, encompassing 15 OR genes, for which deletion alleles were initially identified by qPCR. For 8 out of the 9 deletions we estimated deletion allele frequencies from the number of homozygously deleted individuals in our cohort at approximately 0.2 up to 0.6 (Table 3). Notably, *deletion VII* appears only in the Yoruban individuals, which may suggest population-specificity of this allele. Yet, this bias should be interpreted with caution, as the number of samples we employed from each population is insufficient for meaningful comparison between the populations.

Examination of the chimpanzee reference genome (see Methods) shows that all deletions identified in this study are likely to represent human derived alleles (Table 3), barring presumably infrequent shared polymorphisms. A recent survey [15] on genomic structural variation in different individuals, which identified deletions with an initial resolution in breakpoint assignment of 20–40 kb (and base-pair resolution for several cases in which breakpoint junctions were sequenced), reported variants that are consistent with all deletion alleles reported in our study. This validates our findings and confirms the frequent occurrence of these variants; thus, a considerable number of OR genes are frequently deleted in humans, with homozygous alleles occurring commonly in the population.

## Discussion

We have carried out a high-resolution analysis of CNVs affecting OR gene and pseudogene loci, and identified many OR-related CNVs for which copy-number variability has not been reported previously. In contrast to a previous low-resolution study [19] we observe that CNVs are enriched among evolutionary young ORs as well as pseudogenes. Our results differ from those published by Nozawa et al., probably due to the significant increase in resolution (nearly 2 orders of magnitude, from 50–100 kb to <1 kb), which enabled us to focus on single loci, rather than clusters, and thus to distinguish unaffected OR genes (non-CNVs) from adjacently located affected OR pseudogenes (CNVs). Our analysis suggests that both formation bias and evolutionary constraints have likely shaped the distribution of CNVs in the human OR repertoire. In fact, both biases are difficult to distinguish. For instance, pseudogenes and other repeats are known to be enriched in vertebrate gene deserts [36], which is presumably both due to mutational and selective biases. Also, we defined OR pseudogenes based on the absence of an intact open reading frame in the human reference genome. This may lead to misclassification of some of the intact genes, which may not be functional in reality, due to missense mutations [37] or mutations affecting non-coding regulatory elements. However, both of these confounding factors are presumably affecting only a minority of loci and thus are unlikely to influence our conclusions in relation to a depletion of CNVs affecting OR genes relative to OR pseudogenes.

Furthermore, our data showed a bias for CNV-enriched OR loci to be located between tandemly oriented segmental duplications, which are known to induce NAHR [38]. Besides NAHR, other formation-processes such as non-homologous end-joining (NHEJ; [39]) are likely to play a role in the genesis of CNVs affecting OR loci. In the future, determining the relative contribution of such mutational processes will require the identification of the breakpoint junction sequences of numerous CNVs. In addition to regional biases caused by different mutational processes involved in CNV formation, large CNVs may sometimes span both genes and adjacently located unprocessed pseudogenes. Unprocessed pseudogenes often arise through tandem duplication of OR loci followed by inactivation of the newly generated, neutrally evolving, paralogous loci. This may lead to biases in the frequency at which pseudogenes vary in terms of copy-number, depending on selective constraints acting on adjacent functional loci. Finally, even in the absence of large CNVs such a bias may occur, as CNVs affecting pseudogenes in the proximity of genes may be detrimental due to long-range regulatory effects (e.g., through interfering with non-coding regulatory elements). Furthermore, the enrichment of CNVs among ORs located in close proximity to telomeres/centromeres may also be reflective of CNV formation biases or selective biases. In this regard, human subtelomeric regions are enriched for segmental duplications, and NHEJ and NAHR presumably operate efficiently in those regions [39]. At least for some cases, we were already able to present sequence-based evidence for the likely involvement of NAHR in CNV formation. In particular, we demonstrated that CNVs causing a fusion of tandemly oriented OR genes were presumably formed through NAHR (Figure S9 and [14]). Such events exemplify a potential mechanism for accelerated functional diversification of ORs, where paralogs are originally created with new function or regulation pattern, rather than through the process of sequential duplication and diversion. Consequently it may be hypothesized that large OR subfamilies came to existence through frequent and/or large duplication events, implying that genes from large OR subfamilies will be prone to reside in CNV loci. However, our data showed no obvious correlation between OR subfamily size and averaged variances of *R* (Figure S10). This may partly be due to a confounding factor, namely the reduced sensitivity of microarrays in detecting CNVs within regions sharing very high sequence similarity (see additional discussion bellow); such regions are enriched in the largest OR subfamilies, and our microarrays may have failed to detect CNVs in these.

As discussed above, our high-resolution data helped to clarify that CNVs do not randomly affect genes and pseudogenes, and that for OR genes purifying selection may operate on top of formation biases. In evolutionary terms, CNVs, which are variants *en route* to fixation, have good potential to influence the OR repertoire size. Here, we have presented evidence for an abundance of polymorphic gene loss events affecting the most copy-number variable group of ORs, i.e. a group classified here as evolutionarily “young”. This may point to one possible underlying mechanism for the well-documented diminution of the human OR repertoire as it is reflected in the considerably reduced human OR repertoire size (i.e. 851 ORs) compared with dog and chimpanzee (∼1000 ORs), and with rat and mouse (∼1400 ORs) ([5] and references therein). It should be noted, however, that although these ORs were herein classified as “young” for simplicity, they do not necessarily have to represent recent gene duplicates. In particular, due to the orthology assignments, ORs that underwent deletion or duplication in the chimpanzee genome are also classified as “young” in our study. In contrast, the more “ancient” ORs potentially provide a more stable backbone of the olfactory subgenome, which is less affected by CNVs and also appears to have an overall positive balance between gains and losses. This slight enrichment for gains may imply stronger evolutionary constraints acting on these ORs, as losses are thought to be more detrimental than gains, and genes under purifying selection are more biased away from deletions than from duplications [13].

The identification of 9 deletion alleles, encompassing 15 OR gene loci and present at appreciable frequency, is significant for studies of olfactory function. Previously, functional OR gene inactivation alleles, involving SNPs leading to in-frame stop codons or substitution of conserved amino acids in an otherwise unmodified OR locus sequence, have been reported [6],[37]. Such alleles were subsequently linked to individual human responses to specific odorants, using both *in-vitro* and association study approaches [12]. However, large deletions have not so far been reported among the variants used for genetic association studies. The present identification of a number of unexpectedly frequent deletion alleles (with deletion allele frequencies of up to 0.6), some of which encompass several genes from the same OR subfamily, thus provides additional strong candidates for genetic association studies of human olfaction. To this end, we have recently initiated a CNV genotyping experiment using qPCR for the herein reported *deletion I* (Table 3) against a Caucasian cohort [40] of 94 subjects, phenotyped for olfactory acuity towards eight odorants (unpublished). The results were inconclusive, probably due to the low number of samples and odorants involved. Future association studies will require larger sample sizes and, ideally, a-priori *in-vitro* assessment of ligand specificity for the affected ORs.

Our study also has certain technical limitations. First, while microarrays represent the most cost-effective method for studying CNVs at large scale and high resolution, cross-hybridization limits their specificity and sensitivity in repetitive genomic regions. Cross-hybridization results in averaging of the signal over several loci, and is thus more likely to lead to false negative than to false positive CNV calls when using a stringent cutoff for scoring the arrays. A second potentially confounding factor is the inter-individual sequence variability, i.e. SNPs and small indels, that may affect probe hybridization. Yet, different probes on our arrays are generally ≫1 bp apart from each other, there are dozens of probes for each locus, and the signal is analyzed over all probes mapping to an OR locus. Thus, inter-individual sequence variability in specific probes, typically at the level of 1 SNP per kilobase, is unlikely to considerably affect our CNV calls. Furthermore, our qPCR results indicate that the false-positive rate is relatively low in our microarray experiments, as opposed to a considerable false negative rate, which was expected due to the stringent cutoff applied for scoring the arrays. Third, the comparative nature of our analysis may introduce an overestimation of the frequencies of some CNVs, if the reference sample carries a rare allele. In such cases, the rest of the samples are expected to show only one type of change – gain or loss, across a majority of the samples. Importantly, this would not change the CNV status of the locus (i.e. whether the OR is considered to be copy-number variable or not), and thus did not affect the main conclusions of this study. We nevertheless specifically addressed this issue by calling CNVs independent from the reference individual (see Text S1), an analysis that did not considerably affect our overall CNV counts and did not change the conclusions of our study. Fourth, a considerable fraction of CNVs may represent recurrent, rather than common variants emerging from single mutational events (Table S3). Distinguishing recurrent from common CNVs coherently will become a challenging task that will require breakpoint-resolution data, which is currently available only for few CNVs affecting ORs. Finally, a large portion of CNVs (62%, Table 1) reported in DGV to intersect with OR loci are not observed in our study. This is likely to be, in part, attributed to the relatively low number of samples we analyzed and to false-negative calls in our study, but also to the fact that for most CNVs listed in DGV the size-ranges have been overestimated (in this regard, note the excellent recent survey published in [29]). Furthermore, a parallel survey of CNVs affecting functional OR loci was published while the present paper has been under review [41]. In this study, the authors report a statistical analysis of a subset of CNVs listed in DGV, as well as an experimental validation of CNVs recorded in DGV, which affect a set of 37 OR loci. In agreement with our data, they failed to validate 16 out of the 37 CNVs tested, despite using 50 samples of diverse ancestry. Altogether, these results support the size over-estimation of previous CNV surveys at low resolution ([29],[41]) and stress the relevance of systematic follow-up studies focusing on CNV subsets.

In conclusion, our results emphasize the importance of carrying out genome-wide CNV surveys at high resolution. This is especially important, if one aims to identify events relevant to association studies, which requires the delineation of CNV event nature (i.e. deletion/duplication or complex, common or recurrent), exact CNV boundaries, and CNV population frequencies. Thus our study both provides insights into the evolution of the largest human gene family, and suggests specific targets for subsequent association studies.

## Materials and Methods

### High-Resolution Oligonucleotide Tiling Microarray Design

To determine copy-number variation at OR loci genome-wide and at exon-level resolution, custom high-resolution oligonucleotide tiling microarrays were designed using Nimblegen/Roche technology. The microarrays contained 71,980 different oligonucleotide probes of length 45–85 bp, adjusted in C+G content to yield similar optimum hybridization temperatures [22]. Further, to save experimental costs and make the platform more efficient, multiplex (4-plex) microarrays were used. We designed the probes to cover all 851 OR loci represented in the reference genome (build35), i.e. 388 OR gene loci and 463 OR pseudogene loci including the respective 5′-regions (i.e., 10–20 kb upstream of genes and pseudogenes) and 3′-regions (2 kb downstream of genes/pseudogenes; depiction of probe locations for an example OR locus are presented in Figure S1). Before assigning oligonucleotide probes to loci, we separated the frequently partially overlapping OR loci within genomic gene clusters to avoid assigning probes to multiple ORs loci. Overlapping loci were separated according to the orientation of the OR open reading frames they contained. If both ORs were oriented 5′ to 5′ or 3′ to 3′, the region between them was divided equally between the 2 loci. However, if the orientation was 5′ to 3′, the separation was such that 15% of the region (not exceeding 2 kb) was assigned to the 3′ of one of the OR loci, and 85% were assigned to the 5′ of the other OR locus. Genomic coordinates of OR genes and pseudogenes were obtained from the HORDE database (http://bip.weizmann.ac.il/HORDE/).

A median genomic distance between interrogated oligonucleotides of 148 bp was used on the arrays, a density expected to allow the identification of breakpoints of CNVs at ∼500 bp resolution [23]. Our custom microarray design approach focused on optimum oligonucleotide probes and excluded highly repetitive regions. In particular, (i) oligonucleotides of length 85 bp were extracted from the human reference genome; each oligo was truncated according to its GC content [22] and subsequently aligned against the genome using BLASTN (ncbi); (ii) the array design was initiated by picking oligonucleotides with an offset of 148 bp, thereby considering oligos only if these did not reveal an additional hit to the reference genome at a sequence identity cutoff *S* of 90%, indicating probe redundancy. (iii) If less than the average number of probes per gene/pseudogene locus were picked in the previous step, additional oligos were added to the array, initially by changing the offset to 74 bp, and then by incrementally increasing *S* from 91% to 99%. The median number of probes per locus was 86; in some instances, i.e. in case of highly similar (paralogous) regions in the genome, the minimum number of independent probes was 20 for each OR locus (as an exception, three small OR pseudogene loci were included in the set, having 14, 15, and 16 independent probes, respectively). In 3 cases, to reach a minimum of 20 independent probe measurements per OR locus and facilitate robust CNV detection, duplicate probes were included in the set (as for all other probes used, these duplicates matched perfectly to the target locus only).

### DNA Samples

Twenty-five genomic DNA samples (DNA from cell lines obtained from Coriell; mostly HapMap samples) were used in this study, covering three populations (European, Asian, Nigeria/Yoruba), as follows:

NA12003, NA12004, NA12005, NA12006, NA12246, NA12248, NA12865, NA10851, NA11997- Europeans (CEPH);

NA15510- presumably European [14];

NA18611- Asian (Chinese origin);

NA18945, NA18946, NA18972- Asians (Japanese origin);

NA18504, NA18508, NA18856, NA19103, NA19141, NA18501, NA18502, NA18505, NA18506, NA19128, NA19154- Africans (with origin from Nigeria; Yoruba).

### High-Resolution Oligonucleotide Tiling Microarray Analysis

We interrogated genomic DNA from pairs of two individuals (one labeled with Cy3, one with Cy5, measured in different channels) by following the high-resolution comparative genome hybridization protocol previously described [22]. In our analysis pipeline, Cy3 and Cy5 labeled DNA was independently analyzed – that is, two distinct ‘intensity-measurements’ were retrieved from each panel of the novel Nimblegen multiplex platform (eight distinct measurements per 4-plex microarray experiment; each panel carried 71,980 probes). Quantile normalization (i.e., the algorithm normalize.quantiles from www.bioconductor.org) was used to normalize all channel measurements simultaneously, yielding overall identical intensity distributions for each channel across all experiments. Subsequently, for each oligonucleotide probe on the array, log_2_-ratios *r* were obtained by relating normalized intensities *i* for each probe to median normalized probe intensities calculated across three replicates *c_1_*, *c_2_*, and *c_3_* of a designated control individual (NA19154):
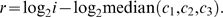



For NA12246, NA15510, NA19103, and NA19141 the microarray experiments were performed in replicate, each replicate was normalized separately. Averaged values of the log_2_-ratios *r* obtained from the replicates were used for further analysis.

CNVs affecting OR loci were called using the following approach: we calculated *R* as the median *r* of all probes that unambiguously map to an OR locus and scored loci as being affected by a CNV if abs(*R*) is greater or equal to a cutoff *C* = 0.18. At this cutoff value, an independent additional technical replicate of NA19541 yielded only 2 predicted CNVs (false positives) affecting OR genes or pseudogenes; this suggests a false positive rate of <4% for our CNV calls, as the mean number of such CNVs in other individuals was 54 (median 53). Since *C* represents a conservative cutoff for *R*-measures that also led us to overlook some true CNVs (see below), we decided to apply raw *R*-values, rather than copy-number calls, for measuring some of the correlations presented below (e.g. such presented in Figures 3, S3, and S6).

### Calculation of Average Number of CNV Events/Sample

Following the scoring of the array data according to cutoff *C* (see above), microarray intensities were converted to discrete values of 1, −1 and 0, representing gains, losses and neutral values respectively. For each sample we counted the total number of CNV events (gains or losses). Next we calculated the average of the total number of events per sample found in 24 individuals.

### Statistical Calculations

Pearson correlations and respective P-values were calculated using Wessa, P. (2008), Free Statistics Software, version 1.1.23-r1, at http://www.wessa.net/. Chi-square tests and contingency tables probabilities were calculated using http://www.physics.csbsju.edu/stats.

### Quantitative PCR

Quantitative PCR (qPCR) was carried out using Absolute Blue SYBR Green Rox Mix (ABgene) on a Roche LightCycler 480. Concentrations of the DNA samples were measured using a NanoDrop 1000 spectrophotometer. The samples were diluted to 1 ng/µl stock. Prior to each experiment, 8 µl of every sample was dispensed into the 96 well qPCR plate by Biomek 3000 robot, in duplicates, and dried over-night. The reactions were carried out in a 96-well plate in 10 µl volume, containing 5 µl of Blue-SYBR-Green mix, 0.5 µM of each primer and 8 ng of genomic DNA. The following thermocycling program was used: Enzyme activation, 95°C, 15 min; 40 cycles of denaturation at 95°C, 15 sec, and annealing & extension at 60 C, 1 min. After amplification the temperature was slowly raised and fluorescence was continuously monitored to produce melting curves of every product, so as to verify product specificity. Reactions that indicated more than one peak in the melting curves, were removed from further analysis. All qPCR results are summarized in Table S1.

The qPCR results were normalized using qPCR results of the regulator of calcineurin 1 (RCAN1) gene (previously Down Syndrome Critical Region 1 gene (DSCR1)), presumed not to vary in copy number in normal individuals[24],[25]. For comparisons between microarray and qPCR results we performed a further normalization by log scale subtraction of the value for the designated reference sample NA19154. The 56 most variable OR loci were selected based on the variance of qPCR results, such that 25 most variable blocks of OR loci were represented. ORs that did not show gains or losses, but revealed a variance in qPCR results >0.15, were considered as additional CNVs.

### Standard PCR and Gel Electrophoresis

Polymerase chain reaction (PCR) amplification was performed in 10 µl volume, using Qiagen HotStart Taq polymerase enzyme, and standard supplier protocol. The following thermocycling program was used: enzyme activation, 95°C, 15 min; followed by 45 cycles of denaturation at 95°C, 15 sec, annealing at 58°C, 30 sec, and extension at 72 C, 1 min. Products were analyzed by gel electrophoresis, in 2% agarose gel with 100 bp marker.

### Primer Design

Primers were designed with Primer3 (http://fokker.wi.mit.edu/primer3/input.htm) software using the following parameters: melting temperatures (Tm) between 56 and 60°C, GC-content between 30% and 70% and length from 18 to 28 bp. Uniqueness of primers and amplicons was checked using BLAT and in-silico-PCR against the hg18 reference assembly (available at http://genome.ucsc.edu) [26]. In the case of qPCR, all primers were designed to amplify 100–200 bp segments. In cases where automatic design failed to produce specific primers we designed them manually using sequence alignments. All primers were adjusted for the thermodynamical properties used in Primer3, and checked for uniqueness as described above.

### Computational Identification of CNV Breakpoints Affecting OR Loci

CNV breakpoints affecting OR loci were identified using two complementary approaches. (*i*) Breakpoints of CNVs were predicted from the high-resolution microarray data using the *BreakPtr* algorithm [23]. Namely, although most CNV breakpoints likely occur outside OR gene/pseudogene loci, we tested whether some CNVs disrupt the loci targeted in this study. We therefore applied the default parameters of *BreakPtr* (‘core parameterization’ [23]) to fine-map CNV breakpoints. We conservatively considered breakpoint-assignments within a locus as robust, if a minimum of 5 probes were present on either side of the breakpoint junction and supported the predicted dosage change [23] (only such robust breakpoint assignments were reported by us). (*ii*) Deletion breakpoints were identified computationally through analyzing genomic clones deposited in Genbank that are not included in the current reference genome. Namely, fosmids were selected from Genbank by using the keywords “fosmid” and “complete sequence”. This library, which contained 1807 clones, was screened specifically for OR genes using the TBLASTN (ncbi) algorithm. Altogether, we found 30 fosmids that contained at least one full-length OR gene. These 30 fosmids were subsequently aligned to the human genome in 20 kb fragments using the BLAT algorithm [26]. Fragments that aligned to regions of lengths different from 20 kb were suspected to contain deletions or insertions, and were inspected manually. The clone AC208786 was found to contain the *OR8U8* and *OR8U9* genes, both of which are not present in the reference genome. AC193144 contains *deletion VI*, and AC210900 contains *deletion I*, listed in Table 3. *OR9G9* was found on chr11 of the Celera assembly, and also on clone AC212901. The Celera assembly was mined using methodologies described in [27]. To identify deleted ORs from Craig Venter's genome [28] we analyzed table “HuRef_homozygous_indels_inversion_gff.txt” from the supplementary material of [28].

### Orthology Assignments

We identified OR loci with a one-to-one ortholog in the chimpanzee genome by comparing OR gene/pseudogene coding regions in a pair-wise and bidirectional fashion between human and chimpanzee using BLAST [26]. OR loci were assumed to have a one-to-one ortholog in chimpanzee if, and only if, a corresponding bi-directional (i.e. reciprocal) best hit was found in the chimpanzee genome. The median protein sequence identity of bi-directional best hits was 98%.

### Determination of Ancestral Alleles

Ancestral alleles for deletions were determined based on bidirectional best hits between the whole deletion region and the individual OR genes versus the *Pan troglodyes* reference assembly (March 2006, Build 2.1). For that purpose, orthologous regions between human and chimpanzee were aligned using MEGABLAST (ncbi) and BLAT. For the *OR8U1* locus we also used a sequence from Celera's latest human genome assembly (R27c). Our searches usually revealed syntenic and highly identical orthologous regions (coverage>98% and maximum identity>96%), except for *OR2T10* (coverage = 82% and maximum identity = 80%), for which the region coincides with a gap in the chimpanzee genome.

## Supporting Information

Figure S1 Example of array design in specific OR region. Array probes are depicted in red, OR territory and open reading frame in blue (5′ and 3′ are marked accordingly),and repeat regions are in black. The depicted image is based on a UCSC browser representation of an exemplary region (OR4C11). Microarrays contained 71,980 different oligonucleotide probes of length 45–85 bp. We designed the probes to cover all 851 OR loci represented in the reference genome (build35), including the respective 5′-regions (10−20 kb upstream), and the 3′-regions (2 kb downstream). The median genomic distance between interrogated oligonucleotides was 148 bp; probes in highly repetitive regions were excluded. Note that due to the resolution, in some cases overlapping probes may appear as thick red lines.
(0.36 MB TIF)

Figure S2 Variance of experimental measures in qPCR and microarray experiments. Mean variance of the qPCR data (upper panel) and the corresponding measure R from the arrays (median normalized log2-ratio of all probes that unambiguously map to an OR locus; here “array intensity”) for 122 OR loci (lower panel). The red dotted line indicates a conservative variance cutoff of 0.15 for qPCR results (ORs with qPCR measures beyond this cutoff presumably represent CNVs and are given in Table 3).
(0.83 MB DOC)

Figure S3 Correlation of qPCR and microarray experiments. A) Correlation of variance in qPCR and array measures R. Each data point represents variance measures for a single OR locus. The correlation coefficient measured for ORs affected by CNVs is C = 0.37 (Pvalue = 0.005), and C = 0.02 (Pvalue = 0.4; not significant) for unaffected ORs. Red dots represent ORs affected by CNVs called with our microarrays; blue dots: unaffected ORs. B) Correlation between normalized qPCR values and array measures R. Each data dot indicates a single individual and its measured values in both qPCR and array experiments for a single OR locus. Correlation for ORs affected by CNVs is C = −0.43 (Pvalue = 10−50), and for unaffected ORs C = −0.08 (Pvalue = 0.001). Linear regression fits of each dataset (CNVs and unaffected ORs) are indicated with red and blue dashed lines, respectively.
(0.72 MB TIF)

Figure S4 Distribution of qPCR variance measures for 122 ORs. A conservative cutoff of 0.15, likely corresponding to a CNV, is indicated by a red dashed line.
(0.57 MB TIF)

Figure S5 Detection of a homozygous deletion of OR4C11 in 2 samples. qPCR amplification curves and PCR gel product for the OR4C11 locus. Samples NA12003, NA12004, NA12246 and NA12248 are labeled 1–4, respectively. Panel I: qPCR amplification curves for OR4C11 in 23 individuals. Black arrows indicate curves corresponding to a negative control (BL); red arrows indicate samples (1,3) with a presumed homozygous deletion. Panel II: Standard PCR results of OR4C11 in the 4 samples, analyzed by gel electrophoresis (see Methods)
(0.95 MB TIF)

Figure S6 CNVs affecting the OR repertoire are multi-locus and associated with telomeres/centromeres. A) Correlation between mean variances in microarray experiments (i.e. R-measures) for each pair of adjacent ORs in the same cluster. The variance for each OR locus (OR1) is plotted against the variance of the neighboring OR locus (OR2). Red and blue indicate copy-number variable and non-variable ORs, respectively. The variability status (CNV, non-CNV) was defined according to OR1. Correlation for ORs affected by CNVs is C = 0.8 (Pvalue = 10−18) and for non-variable ORs C = 0.18 (Pvalue = 10−6). Linear regression fits of each dataset are shown as dashed lines. B) Distance of each OR locus to telomere and centromere. Correlation for ORs affected by CNVs is C = −0.17 (Pvalue = 0.003), and for non-variable ORs C = 0.03 (Pvalue = 0.19; not significant). Linear regression fits of each dataset are shown as dashed lines. C. Pearson correlation of cross-individual patterns based on R between two consecutives variable ORs (see text). For this analysis we chose 140 ORs, from 36 OR clusters, and used the criteria that at least 2 consecutive ORs, of the same cluster, had to be variable.
(1.59 MB TIF)

Figure S7 Distribution of CNV blocks sizes. CNV blocks were defined as adjacent variable ORs (based on R-measures), within the same genomic cluster. Bars show the distribution of the number of ORs in CNV blocks, some of which likely represent large CNVs (see text). Numbers on top of a bar indicate number of CNV blocks for a relevant size.
(0.27 MB TIF)

Figure S8 Overlap between pairs of tandemly oriented segmental duplications (SDs) and genomic OR clusters. Bars indicate average number of SD pairs that overlap with OR clusters. Clusters were classified into 3 groups: “CNV” - clusters for which each OR locus (including genes/pseudogenes) in the cluster was found to be affected by a CNV at some point during our analysis OR-specific, “NV” - non variable clusters for which no copy-number variation was observed, and “mixed CNV/NV” - clusters that encompasses both copy-number variable and non-variable OR loci. Orange bars represent data normalized for cluster size, whereas yellow bars depict results without normalization. Error bars indicate 95% confidence interval, “n” is the number of clusters in each group, “ORs/cluster” indicates average number of ORs in clusters. Note the significant enrichment of segmental duplication pairs in the “CNV”-group compared to the “NV”-group (P-values 0.007 and 0.003).
(0.47 MB TIF)

Figure S9 NAHR affecting the OR8U8/OR8U9 ancestral locus leads to formation of the OR8U1 fusion gene. Schematic representation of the NAHR event in which the two genes OR8U8 and OR8U9 led to the formation of the fusion gene OR8U1. Arrows represent locations of the allele-specific primers we used, demonstrating that both alleles are commonly present in the human population. The bottom panel shows the sequence alignment of the three genes, OR8U8, OR8U9, and OR8U1. Note the highlighted 100% sequence-identity stretch in the alignment of OR8U8 and OR8U1 at the 5′ region (bases 472–590).
(1.35 MB TIF)

Figure S10 Correlation of OR copy-number variability with paralog-similarity, but not with OR subfamily size. OR subfamily assignments were based on the HORDE database (http://bip.weizmann.ac.il/HORDE/). Each circle represents an OR subfamily of certain size, as indicated by circle size and color (according to the scale on the right). Numbers in parentheses indicate numbers of subfamilies of a given size, e.g. there are 52 subfamilies with 2 OR members. The largest subfamily with 85 members is subfamily 7E. Average best-hit % identity was calculated as the average of all % identities of each subfamily member with its closest OR. Average subfamily variance was calculated as the average R-based variance of all ORs in a subfamily. (A linear regression fit was calculated for subfamilies with 2 members.
(0.78 MB DOC)

Table S1 Normalized qPCR Cp values for 122 OR loci. Quantitative PCR (qPCR) data of 122 ORs, measured in 22 individuals. The qPCR values are normalized relative to the RCAN1 gene. NT: -Not Tested-; DEL: reactions consistently failed in both duplicates (in at least two independent experiments), thus indicating a homozygous deletion of the locus.
(0.06 MB XLS)

Table S2 Coordinates and normalized microarray measures R for all OR loci. Mean normalized relative intensities (i.e. the measure R) for 851 OR gene and pseudogene loci, are given as detected using custom high-resolution oligonucleotide tiling microarrays. Genomic coordinates for each locus are indicated. OR loci with R>0.18 were called as gains, and with R<0.18 as losses, respectively, relative to the designated reference individual NA19154.
(0.48 MB XLS)

Table S3 *Evidence for both a gain and a loss observed in a single territory and sample. **This CNV likely corresponds to a deletion with breakpoint junctions fusing OR51A2 and OR51A4, previously seen by us using another method (Korbel et al. 2007, Science 318:420-6). This deletion results in a new gene with a coding region identical to OR51A4 and upstream regions from OR51A2, which is combined with a loss of the OR51A2 coding region. ***Breakpoints within a locus were called if, and only if, at least 5 probes were on either side of the breakpoint junction, supporting the predicted dosage change. ****For eight loci (OR2T11, OR4S1, OR4N2, OR11H3P, OR10H2, OR7E125P, OR7E96P, and OR13J1) at least two breakpoints intersecting with the locus were predicted with a distance of more than 1000 bp apart from each other. This suggests that in those loci CNVs may have been formed recurrently, as the expected resolution of the calls is ∼500 bp (see Methods).
(0.04 MB XLS)

Text S1 Supplementary analysis.
(0.04 MB DOC)
