# Supplementary figures and images for: High-Resolution Copy-Number Variation Map Reflects Human Olfactory Receptor Diversity and Evolution
Source: PLoS Genet. 2008 Nov 7;4(11):e1000249. doi: 10.1371/journal.pgen.1000249 (PMC2570968; doi:10.1371/journal.pgen.1000249)

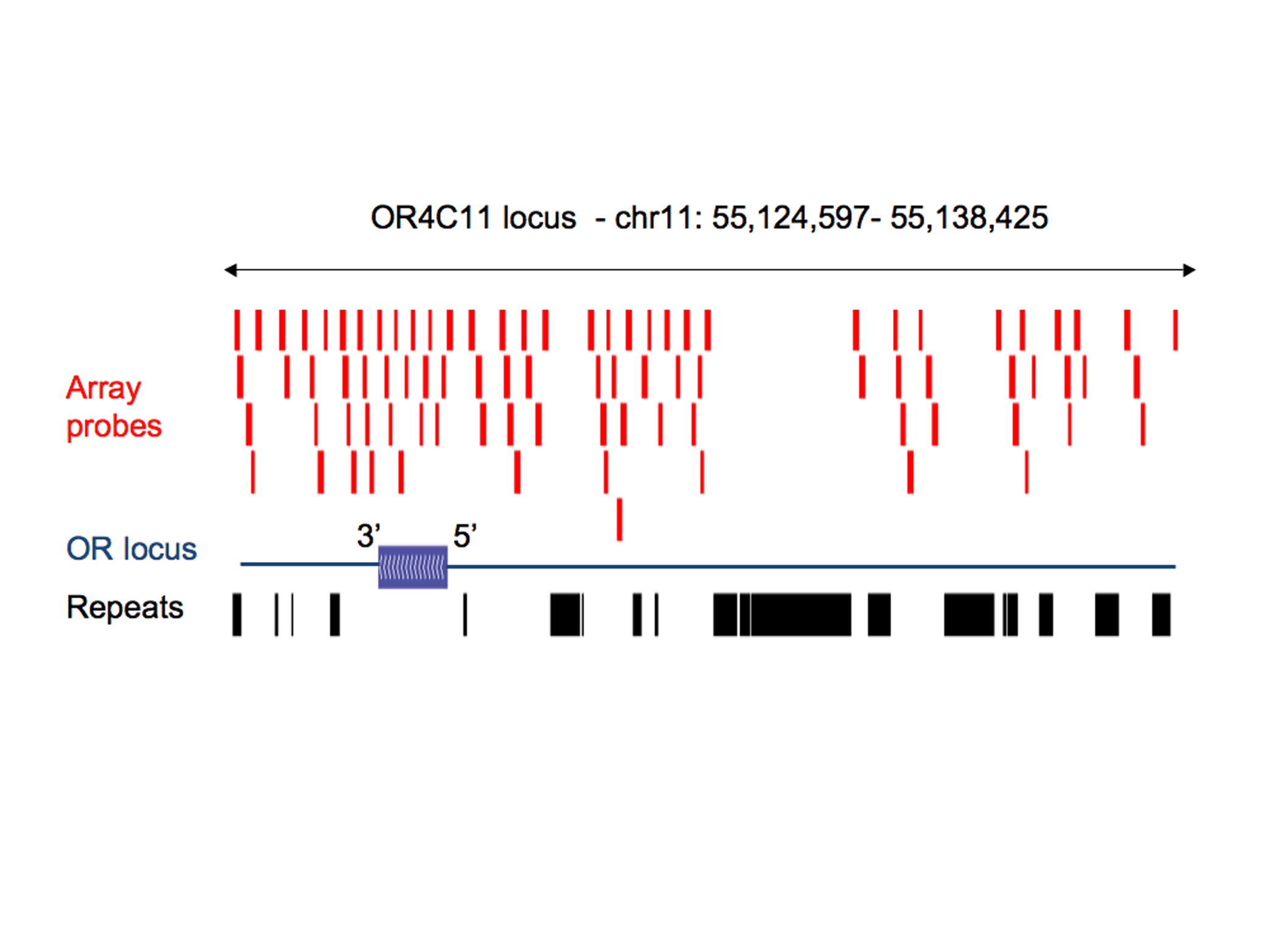

Supplement: Figure S1 — Example of array design in specific OR region. Array probes are depicted in red, OR territory and open reading frame in blue (5′ and 3′ are marked accordingly),and repeat regions are in black. The depicted image is based on a UCSC browser representation of an exemplary region (OR4C11). Microarrays contained 71,980 different oligonucleotide probes of length 45–85 bp. We designed the probes to cover all 851 OR loci represented in the reference genome (build35), including the respective 5′-regions (10−20 kb upstream), and the 3′-regions (2 kb downstream). The median genomic distance between interrogated oligonucleotides was 148 bp; probes in highly repetitive regions were excluded. Note that due to the resolution, in some cases overlapping probes may appear as thick red lines. (0.36 MB TIF) [file pgen.1000249.s001.tif]

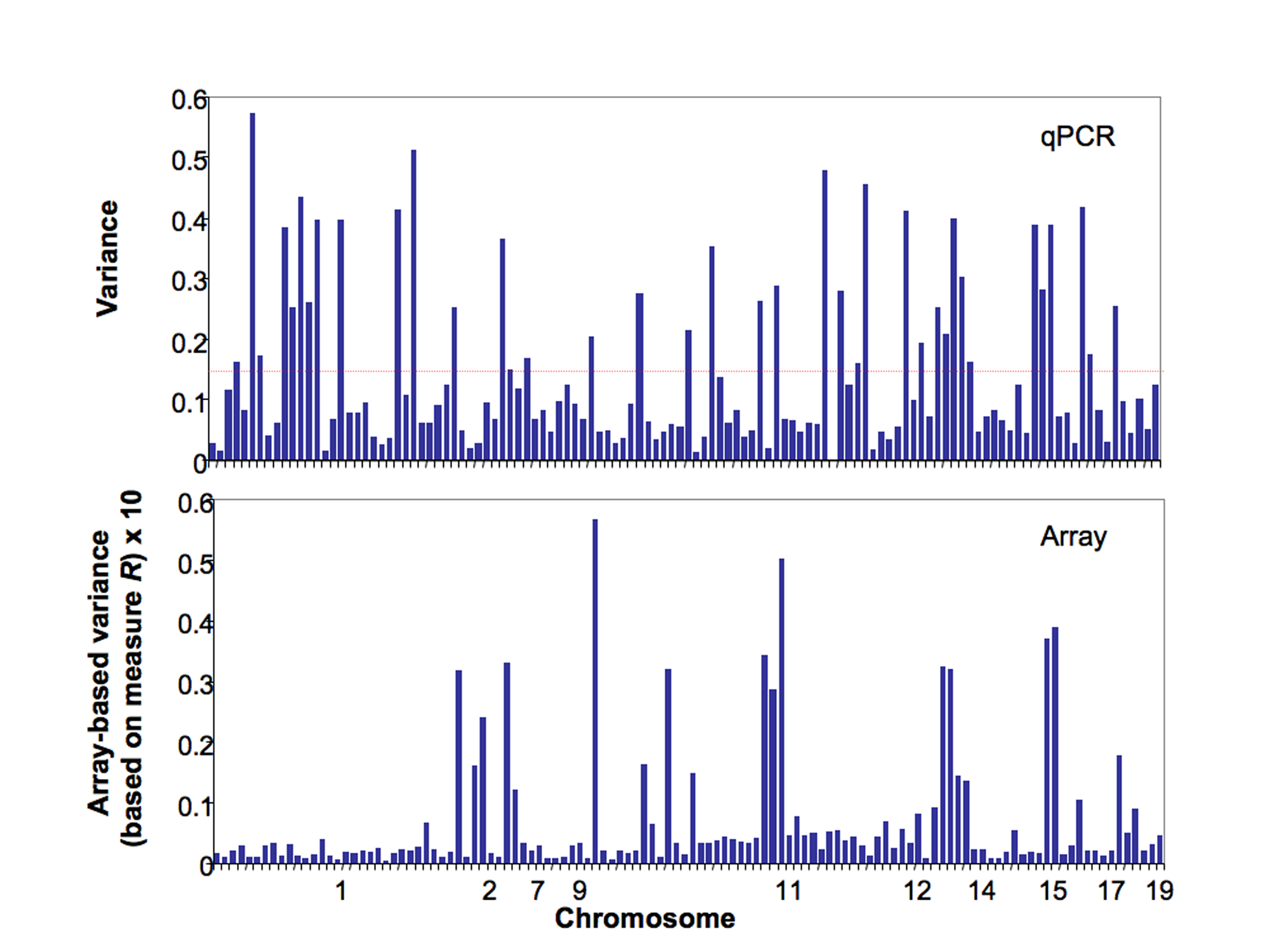

Supplement: Figure S2 — Variance of experimental measures in qPCR and microarray experiments. Mean variance of the qPCR data (upper panel) and the corresponding measure R from the arrays (median normalized log2-ratio of all probes that unambiguously map to an OR locus; here “array intensity”) for 122 OR loci (lower panel). The red dotted line indicates a conservative variance cutoff of 0.15 for qPCR results (ORs with qPCR measures beyond this cutoff presumably represent CNVs and are given in Table 3). (0.83 MB DOC) [file pgen.1000249.s002.doc]

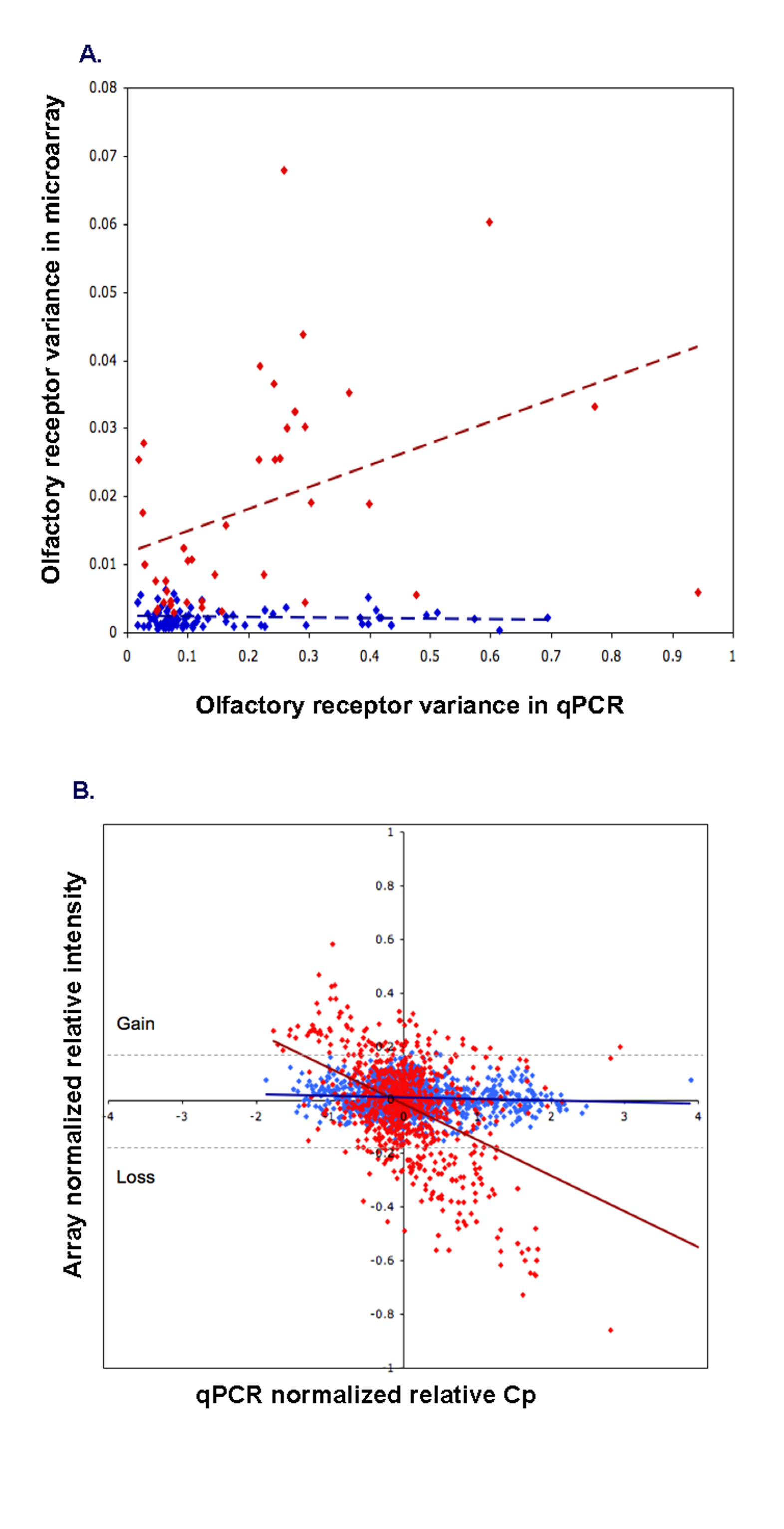

Supplement: Figure S3 — Correlation of qPCR and microarray experiments. A) Correlation of variance in qPCR and array measures R. Each data point represents variance measures for a single OR locus. The correlation coefficient measured for ORs affected by CNVs is C = 0.37 (Pvalue = 0.005), and C = 0.02 (Pvalue = 0.4; not significant) for unaffected ORs. Red dots represent ORs affected by CNVs called with our microarrays; blue dots: unaffected ORs. B) Correlation between normalized qPCR values and array measures R. Each data dot indicates a single individual and its measured values in both qPCR and array experiments for a single OR locus. Correlation for ORs affected by CNVs is C = −0.43 (Pvalue = 10−50), and for unaffected ORs C = −0.08 (Pvalue = 0.001). Linear regression fits of each dataset (CNVs and unaffected ORs) are indicated with red and blue dashed lines, respectively. (0.72 MB TIF) [file pgen.1000249.s003.tif]

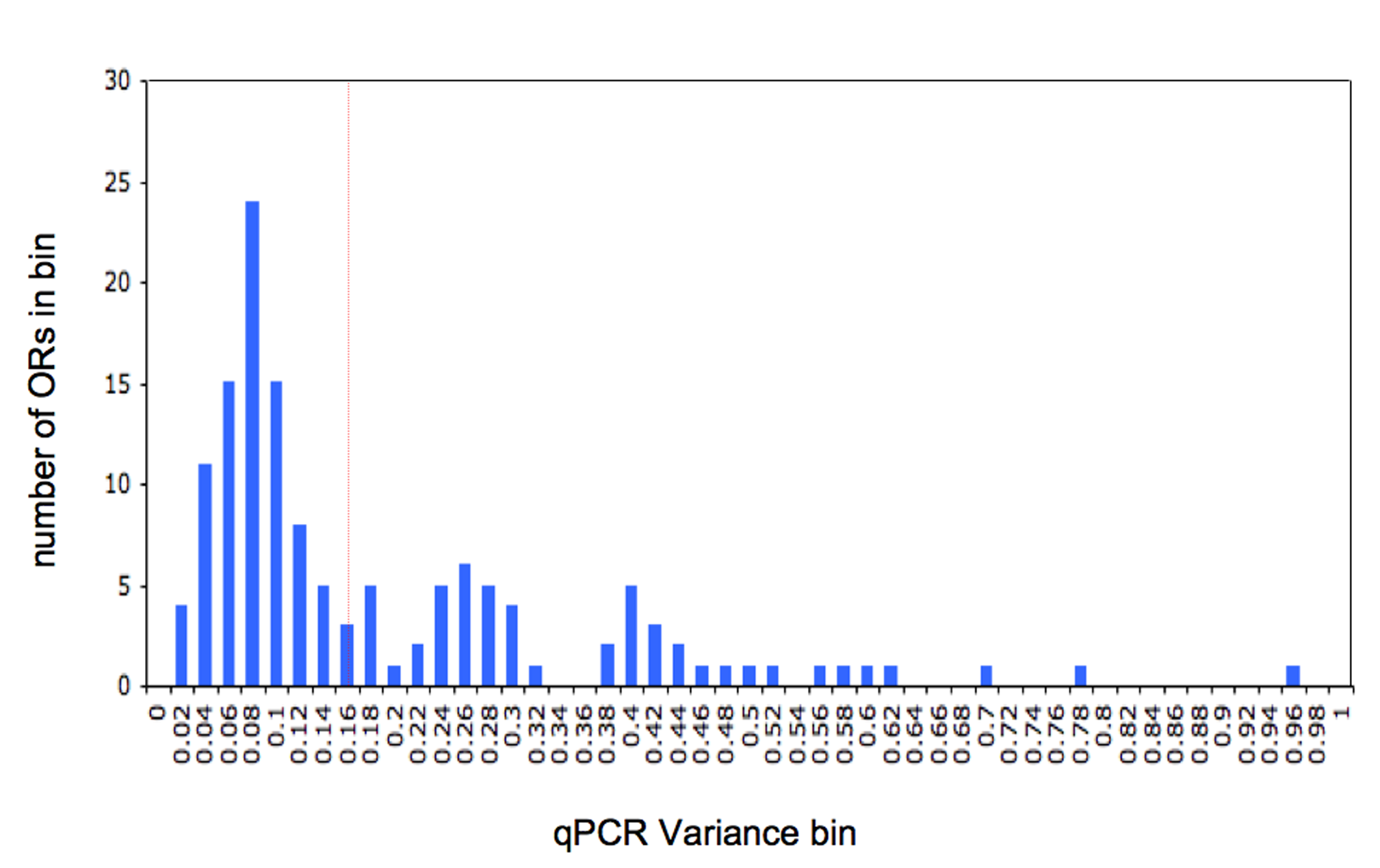

Supplement: Figure S4 — Distribution of qPCR variance measures for 122 ORs. A conservative cutoff of 0.15, likely corresponding to a CNV, is indicated by a red dashed line. (0.57 MB TIF) [file pgen.1000249.s004.tif]

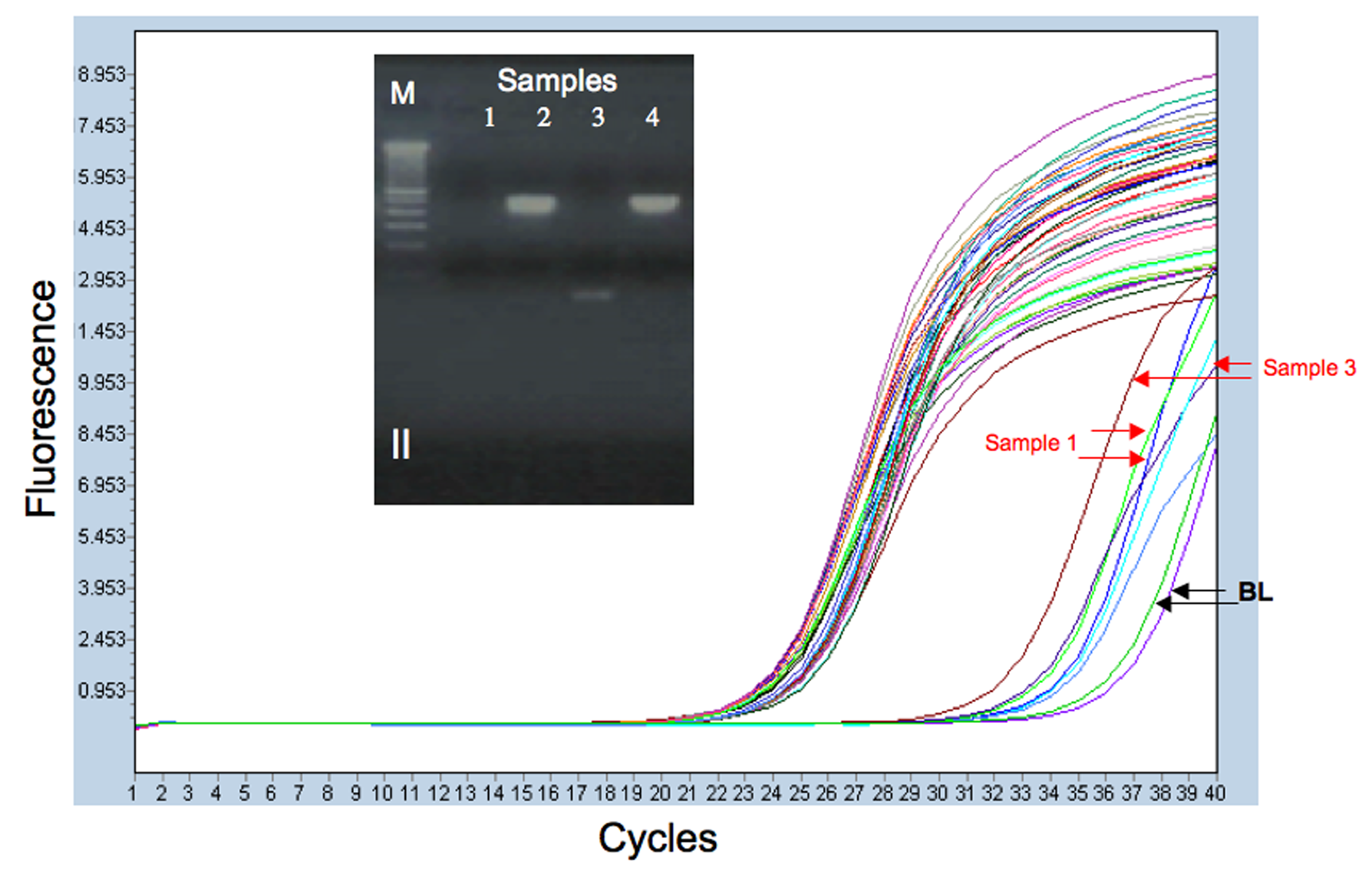

Supplement: Figure S5 — Detection of a homozygous deletion of OR4C11 in 2 samples. qPCR amplification curves and PCR gel product for the OR4C11 locus. Samples NA12003, NA12004, NA12246 and NA12248 are labeled 1–4, respectively. Panel I: qPCR amplification curves for OR4C11 in 23 individuals. Black arrows indicate curves corresponding to a negative control (BL); red arrows indicate samples (1,3) with a presumed homozygous deletion. Panel II: Standard PCR results of OR4C11 in the 4 samples, analyzed by gel electrophoresis (see Methods) (0.95 MB TIF) [file pgen.1000249.s005.tif]

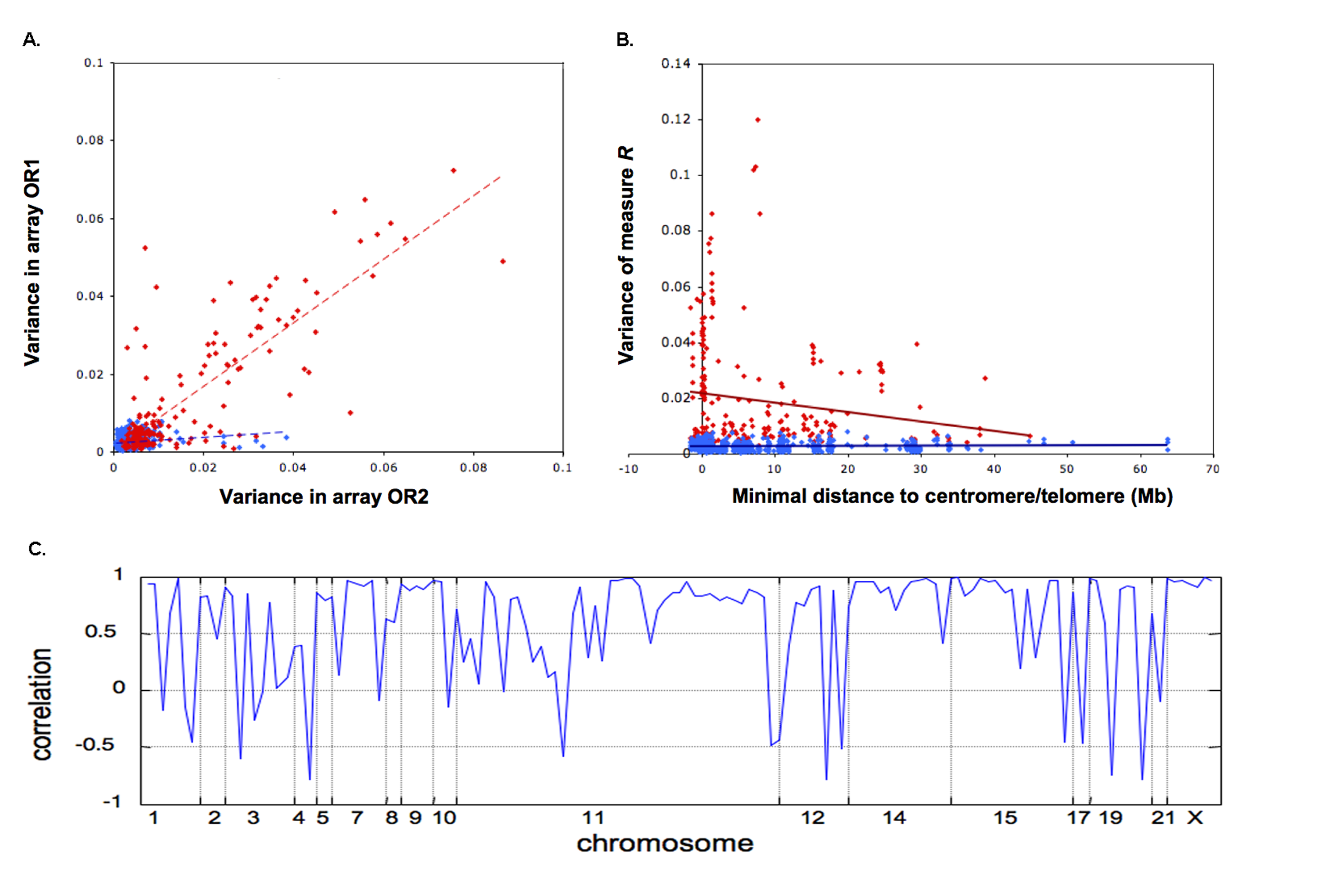

Supplement: Figure S6 — CNVs affecting the OR repertoire are multi-locus and associated with telomeres/centromeres. A) Correlation between mean variances in microarray experiments (i.e. R-measures) for each pair of adjacent ORs in the same cluster. The variance for each OR locus (OR1) is plotted against the variance of the neighboring OR locus (OR2). Red and blue indicate copy-number variable and non-variable ORs, respectively. The variability status (CNV, non-CNV) was defined according to OR1. Correlation for ORs affected by CNVs is C = 0.8 (Pvalue = 10−18) and for non-variable ORs C = 0.18 (Pvalue = 10−6). Linear regression fits of each dataset are shown as dashed lines. B) Distance of each OR locus to telomere and centromere. Correlation for ORs affected by CNVs is C = −0.17 (Pvalue = 0.003), and for non-variable ORs C = 0.03 (Pvalue = 0.19; not significant). Linear regression fits of each dataset are shown as dashed lines. C. Pearson correlation of cross-individual patterns based on R between two consecutives variable ORs (see text). For this analysis we chose 140 ORs, from 36 OR clusters, and used the criteria that at least 2 consecutive ORs, of the same cluster, had to be variable. (1.59 MB TIF) [file pgen.1000249.s006.tif]

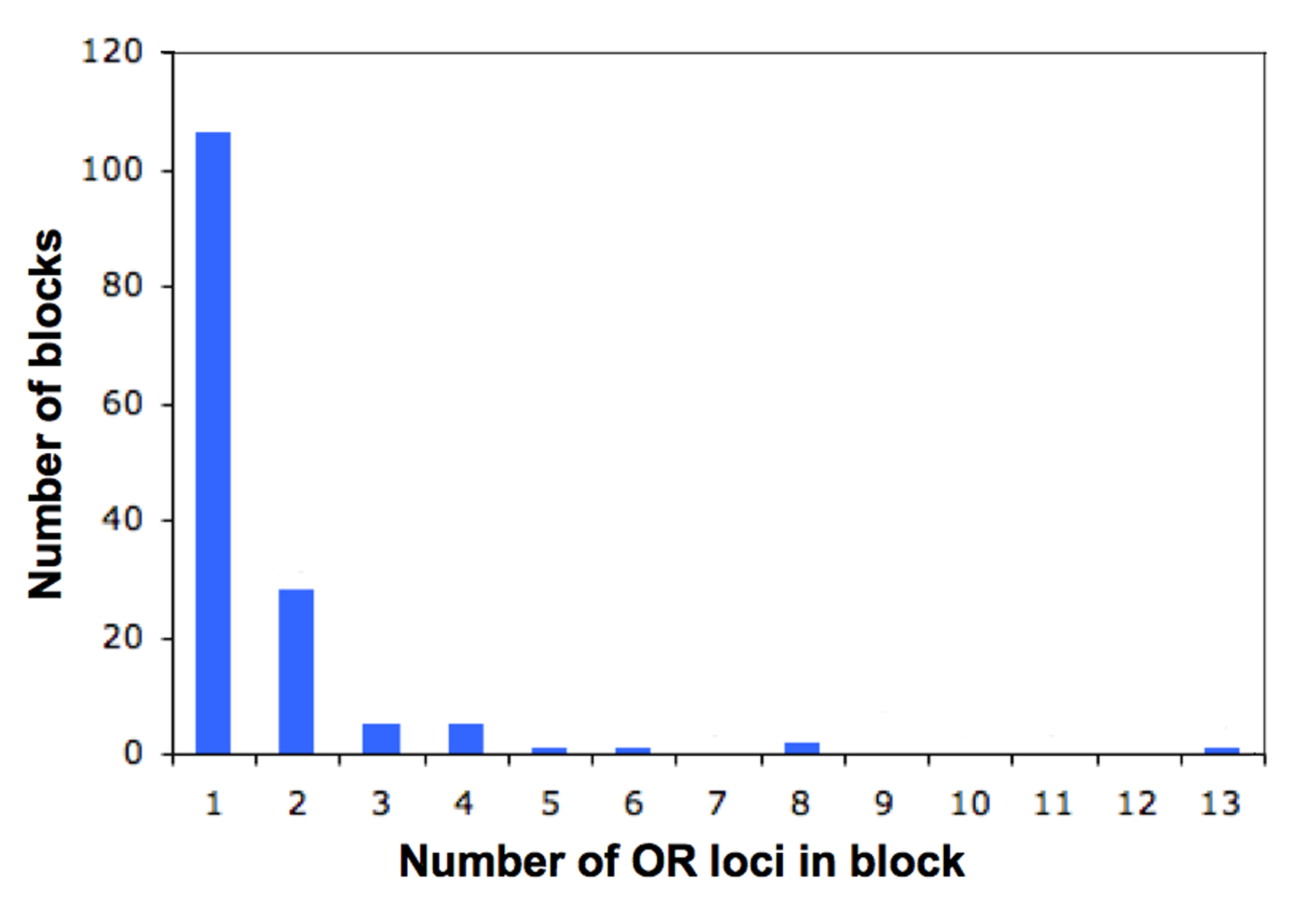

Supplement: Figure S7 — Distribution of CNV blocks sizes. CNV blocks were defined as adjacent variable ORs (based on R-measures), within the same genomic cluster. Bars show the distribution of the number of ORs in CNV blocks, some of which likely represent large CNVs (see text). Numbers on top of a bar indicate number of CNV blocks for a relevant size. (0.27 MB TIF) [file pgen.1000249.s007.tif]

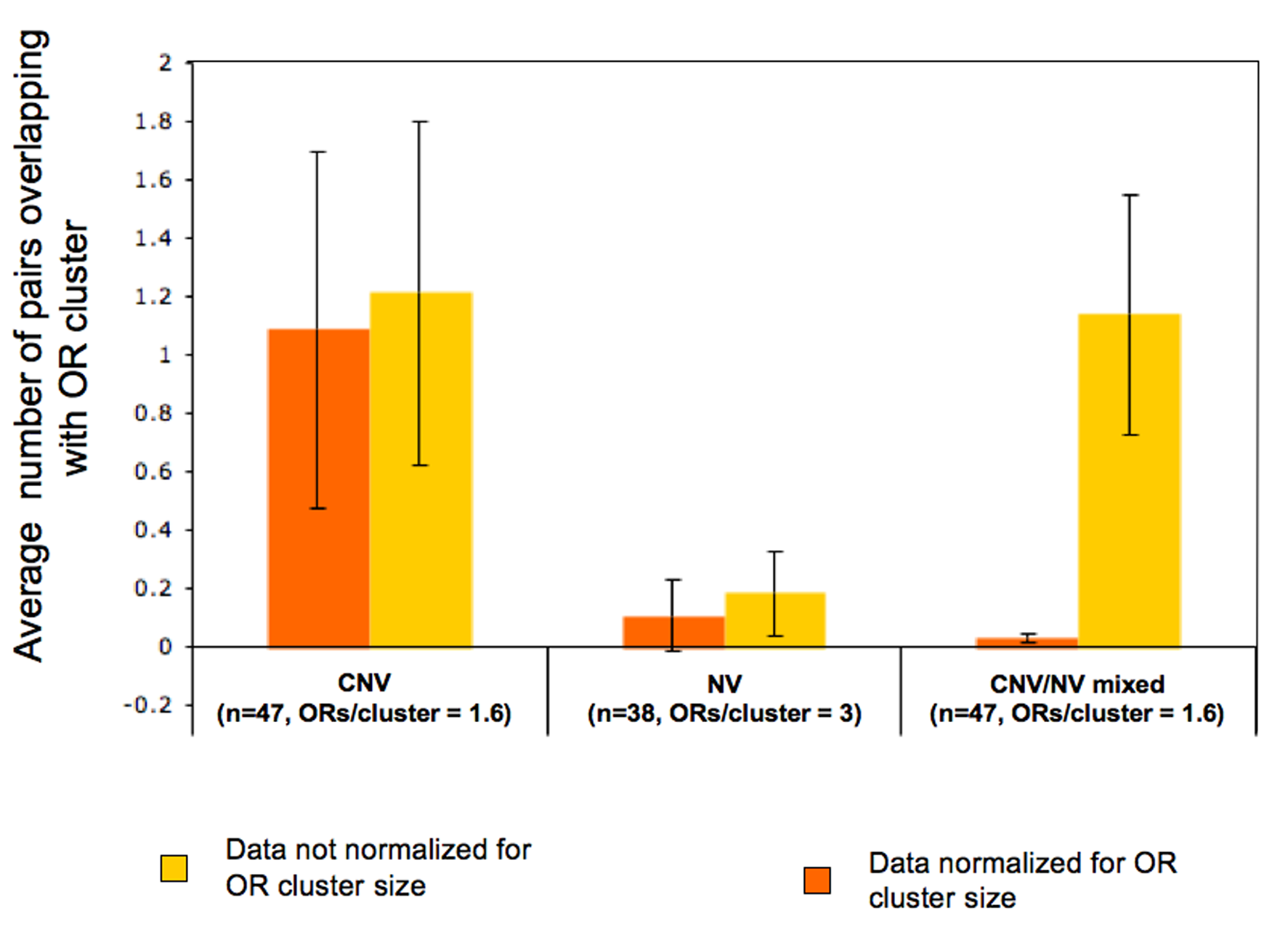

Supplement: Figure S8 — Overlap between pairs of tandemly oriented segmental duplications (SDs) and genomic OR clusters. Bars indicate average number of SD pairs that overlap with OR clusters. Clusters were classified into 3 groups: “CNV” - clusters for which each OR locus (including genes/pseudogenes) in the cluster was found to be affected by a CNV at some point during our analysis OR-specific, “NV” - non variable clusters for which no copy-number variation was observed, and “mixed CNV/NV” - clusters that encompasses both copy-number variable and non-variable OR loci. Orange bars represent data normalized for cluster size, whereas yellow bars depict results without normalization. Error bars indicate 95% confidence interval, “n” is the number of clusters in each group, “ORs/cluster” indicates average number of ORs in clusters. Note the significant enrichment of segmental duplication pairs in the “CNV”-group compared to the “NV”-group (P-values 0.007 and 0.003). (0.47 MB TIF) [file pgen.1000249.s008.tif]

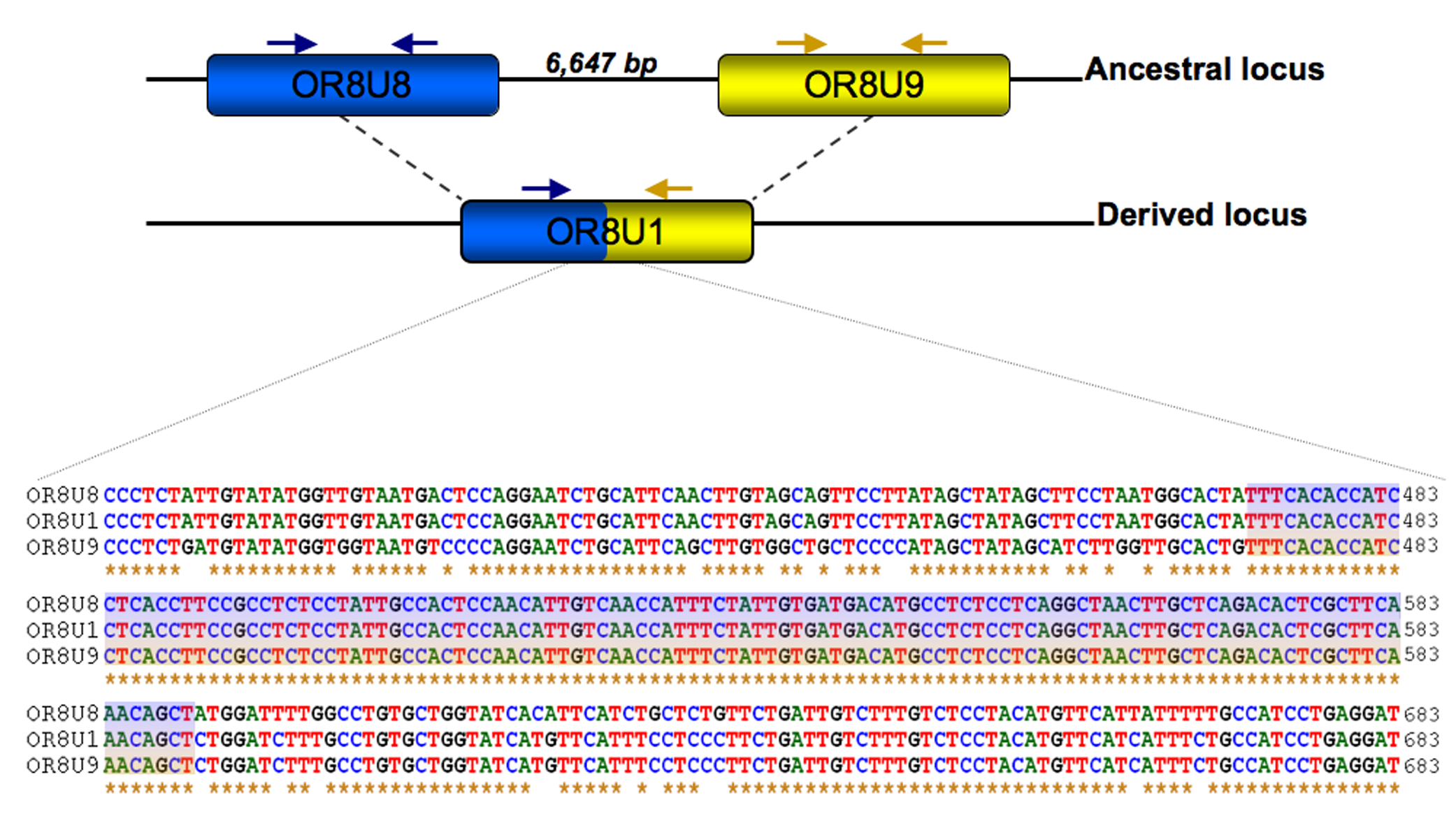

Supplement: Figure S9 — NAHR affecting the OR8U8/OR8U9 ancestral locus leads to formation of the OR8U1 fusion gene. Schematic representation of the NAHR event in which the two genes OR8U8 and OR8U9 led to the formation of the fusion gene OR8U1. Arrows represent locations of the allele-specific primers we used, demonstrating that both alleles are commonly present in the human population. The bottom panel shows the sequence alignment of the three genes, OR8U8, OR8U9, and OR8U1. Note the highlighted 100% sequence-identity stretch in the alignment of OR8U8 and OR8U1 at the 5′ region (bases 472–590). (1.35 MB TIF) [file pgen.1000249.s009.tif]

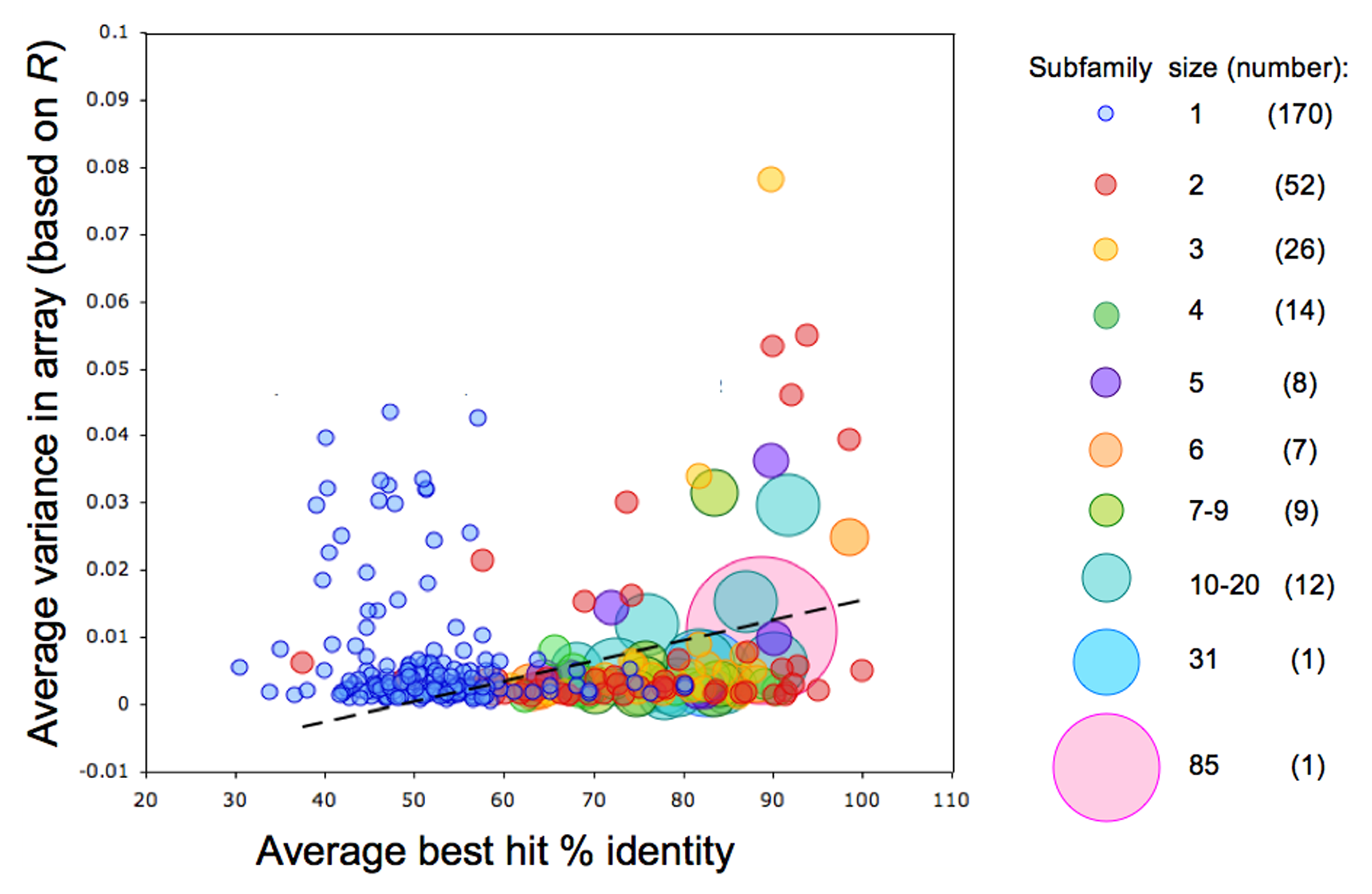

Supplement: Figure S10 — Correlation of OR copy-number variability with paralog-similarity, but not with OR subfamily size. OR subfamily assignments were based on the HORDE database (http://bip.weizmann.ac.il/HORDE/). Each circle represents an OR subfamily of certain size, as indicated by circle size and color (according to the scale on the right). Numbers in parentheses indicate numbers of subfamilies of a given size, e.g. there are 52 subfamilies with 2 OR members. The largest subfamily with 85 members is subfamily 7E. Average best-hit % identity was calculated as the average of all % identities of each subfamily member with its closest OR. Average subfamily variance was calculated as the average R-based variance of all ORs in a subfamily. (A linear regression fit was calculated for subfamilies with 2 members. (0.78 MB DOC) [file pgen.1000249.s010.doc]
