# Supplementary material for: High-Resolution Copy-Number Variation Map Reflects Human Olfactory Receptor Diversity and Evolution
Source: PLoS Genet. 2008 Nov 7;4(11):e1000249. doi: 10.1371/journal.pgen.1000249 (PMC2570968; doi:10.1371/journal.pgen.1000249)
Supplement: Text S1 — Supplementary analysis. (0.04 MB DOC) [file pgen.1000249.s014.doc]

**Supplementary material for manuscript:**

**High-resolution Copy-Number Variation Map Reflects Human Olfactory Receptor Diversity and Evolution**

**Supplementary Analysis**

*Identification of rare de-novo CNV events in the reference sample*: Comparison to a single individual (i.e. NA19154) may inflate the actual numbers of “gains” and “losses”, because if the reference individual has a unique gain it will be reflected as losses in all other individuals and vice versa. To examine the robustness of our analyses, we searched for ORs that exhibited only one type of CNV – gain or loss, in at least 50% of the samples. There were 10 OR loci fulfilling this criterion (OR2A7, OR2A20P, OR2A4, OR2A1, OR2A42, OR2A9P, OR4H6P, OR4H12P, OR4S1, and OR4C6), with 5 of them oriented in tandem in the same cluster (OR2A7, OR2A20P, OR2A1, OR2A42, OR2A9P; in this case, a single large variant in the reference sample NA19154 may have caused the observed copy-number variation). To assess whether CNV calls in these 10 loci may have influenced in any way the analyses or trends reported in this manuscript, we “inverted” the CNV calls for all 10 ORs, to reflect the fact that they may have been caused by a rare allele in the reference individual (for example, OR2A7 showed 23 losses and 0 gains, therefore it was inverted to 2 gains and 0 losses, respectively). After this adjustment the total count of CNV events changed only slightly to 1169 (versus 1301 before). Importantly, this type of correction did not influence whether a specific locus was considered to be copy-number variable or not, since loci were generally considered as variable if displaying at least one gain or loss in any of the samples. Thus all significant trends reported in the main text remained unaffected, except for Fig. 5A, which specifically refers to gain and loss balance in “young” and “ancient” OR loci. In this regard, the trend of opposite balance between the “young” and the “ancient” groups remained significant (P_value_=0.004, χ^2^ statistic = 11.1, DF=2). However, the intra-group (within “young” or “ancient”) differences between gains and losses became less pronounced and lost statistical significance.

*Analysis of segmental duplications in copy-number variable ORs*: Besides selection, locus-specific differences in CNV formation may be the cause for biases in the distribution of CNVs among OR loci. We assessed the effect of CNV formation-bias in our OR set through searching for genomic signatures that are consistent with a common mechanisms involved in CNV-formation, i.e. non-homologous recombination (NAHR). In particular, pairs of tandemly oriented intra-chromosomal segmental duplications (SDs) are known to promote NAHR, and the non-uniform genomic distribution of such SD-pairs may result in local CNV-formation biases [1]. In order to estimate whether the observed differences in copy-number variation among OR loci are attributable to NAHR, we tested for the enrichment of SD pairs among copy-number variable OR loci. For that purpose, we first extracted the genomic coordinates of pairs of tandemly oriented SDs from the Segmental Duplication Database (humanparalogy.gs.washington.edu/build36). In particular, we only used SD pairs with inter-pair distance of 1 kb-1Mb, and a minimum sequence identity of 90% between the duplicons – reasonable parameter selections in the light of the size-ranges of most CNVs listed in DGV (<http://projects.tcag.ca/variation/>), and of current assumptions about the predisposition of a genomic region towards NAHR (see [2]). We then calculated the number of SD pairs that fully or partially overlap each OR locus. Subsequently, we analyzed the enrichment of SD pairs among OR loci affected by CNVs versus non-variable loci. When performed at single gene resolution, this analysis yielded inconclusive results (i.e. it did not result in any statistically significant outcome). Nevertheless, OR loci tend to be closely located in the genome and often appear adjacent to each other (in fact, the 851 human OR loci have been categorized into 135 genomic clusters by the HORDE database; <http://bioportal.weizmann.ac.il/HORDE/index.html>).Thus, a gene-level analysis is likely to be confounded through a strong positional bias, which will be particularly pronounced within large genomic OR locus clusters containing both copy-number variable and non-variable loci. Therefore, we repeated the analyses for genomic OR locus clusters, arguing that such an analysis is likely to reveal more robust results. For that purpose, we initially divided the 135 OR clusters into three *groups*: “CNV”-clusters for which *each* OR locus (genes and pseudogenes) within the cluster was *at least once* found to be copy-number variable in our analysis (47 clusters); “NV”-clusters for which no copy-number variation was observed at all (38 clusters); and “mixed CNV/NV”-clusters encompassing a mix of variable and non-variable loci (50 clusters). As reflected by the mean number of ORs per cluster (Fig S11), our classification results in a bias of “CNV” and “NV” groups towards relatively small clusters sizes, whereas the “mixed CNV/NV”-clusters were larger on average. Note that by using this grouping we did not attempt to discriminate between single large CNVs spanning all loci of a cluster and several overlapping small CNVs from different individuals that when combined together cover an entire genomic OR cluster. Note further that one potential caveat of such an analysis is that large clusters may exhibit an enrichment in SD pairs merely due to their size. To exclude that this confounding effect may affect our comparison, we carried out the enrichment analysis in two different ways. First we initially compared the *mean SD-pair counts per cluster* among “CNV”, “NV”, and “mixed CNV/NV”-clusters. Second, we compared the *mean normalized SD-pair counts* among the three cluster groups, where the normalization involved scaling the count of SD pairs for each cluster by the length of the cluster in kilobases (Fig S8). Interestingly, both comparisons revealed a significant enrichment of SD-pairs among the “CNV”-clusters relative to the “NV”-clusters (Figure S8; t-test P-values 0.007 and 0.003, respectively), whereas results for the “mixed CNV/NV” group remained inconclusive (no significant outcome observed). This suggests that to some extent NAHR causes CNV-formation biases influencing a subset of genomic OR locus clusters.

*Probe-level comparison of gene and pseudogene loci:* To assess the robustness of our findings that OR pseudogenes are more likely to be affected by CNVs than OR genes, we also compared OR genes and pseudogenes at the level of microarray probe signals *r*. In particular, we compared microarray probes that fall into the coding regions of OR genes to the corresponding regions of OR pseudogenes, and found that OR pseudogenes displayed a significantly increased variance of *r* (variance increased by 3.3%; P<1e-14; Mann-Whitney U test). Furthermore, we repeated this analysis, by comparing values of *r* of probes falling into the combined non-coding upstream/downstream regions of an OR locus with probes falling into the corresponding regions of OR pseudogenes: again, OR pseudogenes displayed a significantly increased variance of *r* (variance increased by 3.6%; P<0.01; Mann-Whitney U test). These results are consistent with our findings reported in the main text, i.e. that pseudogenes are overall more affected by CNVs than OR genes.

**References of the Supplementary Material:**

1. Lupski JR (2007) Genomic rearrangements and sporadic disease. Nat Genet 39: S43-47.

2. Tuzun E, Sharp AJ, Bailey JA, Kaul R, Morrison VA, et al. (2005) Fine-scale structural variation of the human genome. Nature Genetics 37: 727-732.
